# The New Era of Cancer Immunotherapy: Targeting Myeloid-Derived Suppressor Cells to Overcome Immune Evasion

**DOI:** 10.3389/fimmu.2020.01680

**Published:** 2020-07-30

**Authors:** Paola De Cicco, Giuseppe Ercolano, Angela Ianaro

**Affiliations:** ^1^Department of Pharmacy, School of Medicine, University of Naples Federico II, Naples, Italy; ^2^Department of Pathology and Immunology, University of Geneva, Geneva, Switzerland; ^3^Ludwig Institute for Cancer Research Lausanne, University of Lausanne, Lausanne, Switzerland

**Keywords:** immune evasion, melanoma, breast cancer, hepatocellular cacinoma, non-small cell lung cancer (NSCLC), myeloid derived suppressor cell (MDSC), prostate cancer, colorectal cancer

## Abstract

Suppression of antitumor immune responses is one of the main mechanisms by which tumor cells escape from destruction by the immune system. Myeloid-derived suppressor cells (MDSCs) represent the main immunosuppressive cells present in the tumor microenvironment (TME) that sustain cancer progression. MDSCs are a heterogeneous group of immature myeloid cells with a potent activity against T-cell. Studies in mice have demonstrated that MDSCs accumulate in several types of cancer where they promote invasion, angiogenesis, and metastasis formation and inhibit antitumor immunity. In addition, different clinical studies have shown that MDSCs levels in the peripheral blood of cancer patients correlates with tumor burden, stage and with poor prognosis in multiple malignancies. Thus, MDSCs are the major obstacle to many cancer immunotherapies and their targeting may be a beneficial strategy for improvement the efficiency of immunotherapeutic interventions. However, the great heterogeneity of these cells makes their identification in human cancer very challenging. Since both the phenotype and mechanisms of action of MDSCs appear to be tumor-dependent, it is important to accurately characterized the precise MDSC subsets that have clinical relevance in each tumor environment to more efficiently target them. In this review we summarize the phenotype and the suppressive mechanisms of MDSCs populations expanded within different tumor contexts. Further, we discuss about their clinical relevance for cancer diagnosis and therapy.

## Introduction

Cancer immune surveillance is an important process by which the immune system can eliminate nascent tumor cells and to control tumor evolution. Eventually, due to the genetic instability, new tumor cell variants can become resistant to immune effector cells by decreasing their immunogenicity and/or secreting and recruiting immunosuppressive factors in the tumor microenvironment (TME). During this phase of equilibrium, if the immune system is unable to eliminate these clonal variants, then tumors evolve mechanisms to escape from the immune attack allowing malignant progression (1, 2). These mechanisms are diverse but primarily induce attenuation of anti-tumor CD8^+^ T lymphocyte. Immunosuppressive myeloid cells, including myeloid-derived suppressor cells (MDSCs) are key mediators in assisting tumors to escape immune surveillance, contributing to tumor development and progression. MDSCs are a heterogeneous group of immature myeloid cells (IMCs) with strong immunosuppressive patterns and functions. Under normal condition, IMCs quickly differentiate into mature granulocytes, macrophages, or dendritic cells (DCs) which play essential roles in host defense against pathogens. However, in a variety of pathologic conditions such as inflammation, cancer and infection IMCs fail their normal differentiation and acquire the features of immature and dysfunctional myeloid population, which include MDSCs (2). Recently, it has been introduced the hypothesis that MDSCs could also be derived from mature myeloid cells such as monocytes and neutrophils in cancer settings (3, 4). In particular, it has been demonstrated that CD14^+^ cells exposed to extracellular vesicles (EVs) (containing proteins, lipids, and genetic material) isolated from melanoma cells, show a suppressive activity on T cells thus referred as EV-MDSCs. Similarly, it has been reported that the treatment of healthy donor-derived monocytes with chronic lymphocytic leukemia (CLL) cells-derived exosomes induced MDSCs functional characteristics on monocytes mainly driven by miRNA-155 (5). Thus, deregulated myelopoiesis is a common occurrence in cancer and it is accompanied by a reciprocal decline in the quantity/quality of the lymphoid response (6). Myelopoiesis is a tightly controlled process. Certain transcription factors, such as CCAAT/enhancer binding protein-α (C/EBPα), and interferon regulatory factor-8 (IRF-8), are instrumental for normal myeloid cell development, differentiation and function and they can be targets of tumor-derived factors (TDFs). Thus, such TDFs may impair their expression, which ultimately affect the fate of the resulting myeloid response. Indeed, interventions that target atypical myelopoiesis by enhancing IRF-8 expression demonstrated to abrogate MDSC-mediated immunosuppression and to promote MDSCs differentiation in effector myeloid cells including DCs and mature neutrophils with anti-tumor activity (7–9). About 10 years ago, two major subsets of MDSCs have been identified based on their phenotypic and morphological features: monocytic-MDSCs (M-MDSCs) and granulocytic-MDSCs (G-MDSCs). G-MDSCs are phenotypically and morphologically similar to neutrophils, whereas M-MDSCs are similar to monocytes (10). In tumor-bearing mice these cells are characterized by the expression of CD11b and Gr-1 surface markers. The granulocyte marker Gr-1 includes the isoforms Ly6C and Ly6G, and these subsets can be more accurately identified based on their expression as CD11b^+^Ly6C^hi^Ly6G^−^ (M-MDSCs) and as CD11b^+^Ly6C^lo^Ly6G^+^ (G-MDSC) (11). However, several other cell surface markers are introduced such as F4/80, CD124 (IL-4Rα), CD115 (M-CSF-1R), and CD80 (B7.1), which are used for identification of MDSCs subsets and to distinguish MDSCs from neutrophils and monocytes (2, 12). In cancer, the frequency of G-MDSCs in the peripheral lymphoid organs is higher than M-MDSCs. In contrast, MDSCs in tumor sites are mainly M-MDSCs (13, 14). MDSCs are generated in the bone marrow from myeloid progenitor cells and then traffic through the circulatory system into solid tumors. The accumulation of MDSCs in TME mainly depends on two groups of signals. The first group include factors that are mainly secreted by tumor cells, such as stem cell factor (SCF), granulocyte-macrophage colony stimulating factor (GM-CSF), granulocyte colony stimulating factor (G-CSF), vascular endothelial growth factor (VEGF), macrophage colony-stimulating factor (M-CSF). These factors stimulate myelopoiesis and promote the expansion of MDSCs in lymphoid organs and TME by activating the Janus kinase (JAK)-signal transducer and activator of transcription (STAT) signaling pathways. In particular, the transcriptional factors/regulators STAT3, STAT5, IRF8, C/EBPβ, NOTCH play a major role in this process. The second kind of signals includes inflammatory cytokines and chemokines, produced mostly by the tumor stroma, such as IFN-γ, IL-4, IL-6, IL-1β, and CXCL1, which are responsible of inducing the suppressive activity of MDSCs via NF-κB, STAT1, and STAT6 (10, 15). Studies focusing on the role of MDSCs in cancer progression showed that the main activity of these cells is to suppress immunity by perturbing both innate and adaptive immune responses. In tumors, MDSCs have been demonstrated to inhibit cytotoxic T cells proliferation and activation leading to the failure of the anti-tumor immune response, promotion of cancer progression and chemoresistance (16). The main mechanisms implicated in MDSCs-mediated immune suppression include: (i) deprivation of T cells from essential amino acids; (ii) decreased expression of l-selectin by T cells; (iii) induction of oxidative stress; (iv) induction of immunosuppressive cells like regulatory T (T-regs) and T helper (Th) 17 cells (16, 17). Although the role of MDSCs as potent inducers of T-regs has been widely described in different types of cancer, recent findings also demonstrate that T-regs control MDSCs differentiation and function through different molecules such as transforming growth factor (TGF)-β and the programmed death ligand 1 (B7-H1) (18, 19). However, more research is needed to better dissecting the cross-talk between MDSCs and T-regs in the TME. In addition to suppression of immune surveillance, MDSCs can also directly promote tumor progression and metastasis through non-immunological functions by affecting the remodeling of the TME and tumor angiogenesis via production of VEGF, bFGF, Bv8, and matrix metalloproteinase (MMP)-9 (20). The main factors implicated in MDSC-mediated immune suppression include arginase 1 (ARG1), inducible nitric oxide synthase (iNOS), TGF-β, IL-10, cyclooxigenase-2 (COX-2), indoleamine 2,3-dioxygenase (IDO) and many others. M-MDSCs and G-MDSCs can utilize different mechanisms to suppress immune response. M-MDSCs express high levels of ARG1 and of iNOS, thus, they suppress T-cell responses, both in antigen-specific and non-specific manners, trough high production of nitric oxide (NO) in the TME. On the other hand, G-MDSCs are capable of suppressing immune responses primarily in an antigen-specific manner and they act mostly through production of high levels of reactive oxygen species (ROS) (14, 21). Several evidences suggest that on a per cell basis M-MDSC are more potent than G-MDSC (13). In contrast to murine models, the phenotype of MDSCs in humans is not as well-defined. Tipically, human tumor infiltrating MDSCs express the markers CD33 common to cells of myeloid lineage, but lack the expression of the maturation myeloid marker HLA-DR. Analogously to the murine MDSCs, human MDSCs are broadly classified into two different subsets, monocytic and granulocytic, based on the expression of the monocyte differentiation antigen CD14 and the mature monocyte marker CD15. Thus, human M-MDSCs are mostly CD33^+^CD11b^+^CD14^+^HLA-DR^−/low^ whereas human G-MDSCs are CD33^+^CD11b^+^HLA-DR^−/low^CD14^−^CD15^+^. However, the gating strategies used to identify MDSCs populations can vary among researcher. G-MDSCs and neutrophils share similar phenotype; however, they have different density. Recently, identified lectin-type oxidized LDL receptor 1 (LOX-1) allows for better distinction between human neutrophils and G-MDSC. Immune suppressive LOX-1^+^ cells represent 4–15% of all neutrophils in blood of cancer patients and up to 40% in tumor tissues, whereas in healthy individuals, these cells represent <1% (22). Conversely, human M-MDSC can be easily separated from monocytes based on the expression of MHC class II molecules which is expressed only on monocytes (HLA-DR^+^) (23). In addition to the granulocytic and monocytic subtypes, a third small population of putative MDSCs that includes cells with colony-forming activity and promyelocytic appearance was described in humans. These cells, termed immature or early-stage MDSCs (e-MDSCs), have the phenotype CD33^+^CD11b^+^HLA-DR^−^CD14^−^CD15^−^ cells (11, 24). Human M-MDSCs and G-MDSCs, like murine MDSCs, have been shown to exhibit distinct functional attributes. In particular, G-MDSCs primarily use ROS as the mechanism of immune suppression whereas M-MDSCs show up-regulation of iNOS, ARG1, and an array of immunosuppressive cytokines (17). In recent years, the clinical role of MDSCs has emerged. Numerous studies have reported the expansion of MDSCs in various human cancers including breast, colon, lung, pancreatic, renal, esophageal, and melanomas (24–26). Moreover, the frequency of MDSCs have also been negatively correlated with the response to immunotherapy (27). Therefore, targeting MDSCs in cancer patients may be a viable therapeutic approach to reverse immune escape and to maximize immune based treatments.

However, an important issue in this viewpoint is the great heterogeneity of these cells, which make the identification and isolation of human MDSCs subsets very challenging. Several data found a significant diversity in the MDSCs subsets in different human cancers. Moreover, the frequency and the mechanisms of action of each MDSCs subset seems to be influenced by the cancer type (26). Thus, the precise identification of cell surface markers and the exact definition of human MDSCs in different types of malignancies can be useful to improve the efficacy of immunotherapeutic interventions and cancer treatment. In this review, we summarize the phenotype and the biological function of MDSCs populations expanded within different tumor contexts which have showed the strongest negative association with MDSCs, as well as discuss their clinical relevance for cancer diagnosis and therapy.

## Main Strategies to Therapeutically Target MDSCs in Cancer

**Figure 1:**
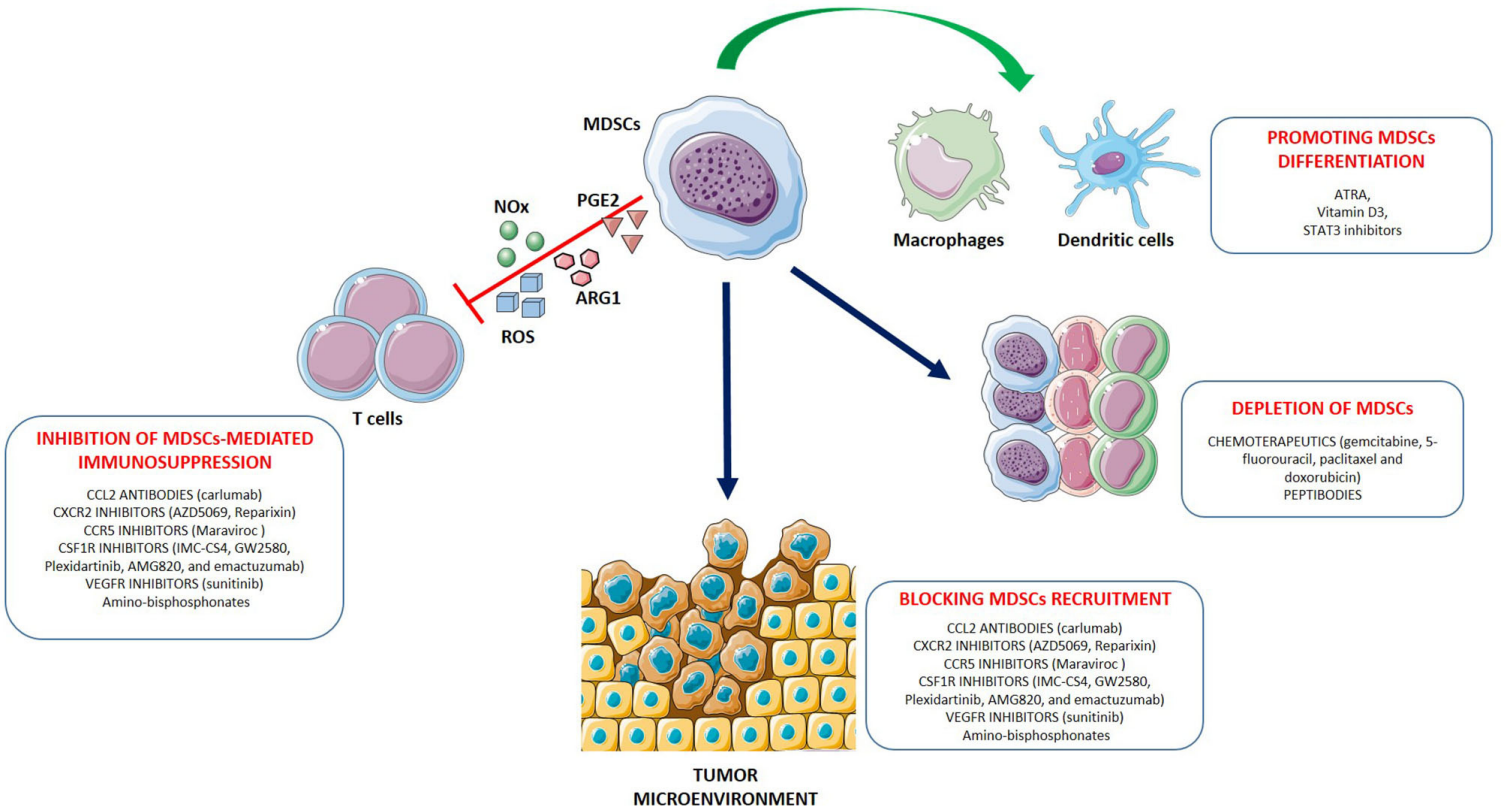
Strategies for myeloid-derived suppressor cells (MDSC) targeting. The main approaches to target MDSCs include: (1) depleting MDSC populations; (2) preventing MDSC recruitment and migration to the TME; (3) attenuating the immunosuppressive mechanisms of MDSCs by downregulating the expression of ARG1, iNOS, COX-2 and reducing ROS generation; (4) promoting the differentiation of MDSCs into mature non-suppressive myeloid cells like macrophages and dendritic cells. Examples for each therapeutic approach are shown.

Inhibition of MDSCs in cancer therapy has proven to be a potentially promising and well-tolerated treatment. Increasing numbers of pre-clinical studies and clinical trials have been performed over the past years in order to evaluate the safety and the efficacy of MDSCs inhibition, alone or in combination with other therapy (radiotherapy, chemotherapy, surgery or immunotherapy) in cancers. Currently, different therapeutic strategies aimed at eliminating MDSCs and/or abrogating their pro-tumor activities are being investigated. These approaches include (1) depletion of MDSCs; (2) inhibition of MDSCs recruitment to the tumor site; (3) inhibition of MDSC's suppressive activity; (4) promoting MDSCs differentiation (Figure 1).

In mouse models, depletion of MDSCs has been generally accomplished by the use of antibodies that target the surface markers Gr-1 or Ly6G (28). More recently, novel approaches have been developed to more preferentially target and deplete MDSCs. For example, “peptibodies” consisting of S100A9-derived peptides conjugated to antibody Fc fragments have shown potential in eliminating MDSCs in mouse models without targeting other proinflammatory immune cells (29). In addition, induction of Fas-FasL mediated apoptosis of MDSCs have been resulted effective in suppressing tumor growth and restoring T cells immune response in different murine tumor models (30–32). Similarly, targeting the TNF-related apoptosis-induced ligand (TRAIL) receptor could be a potent and selective method for MDSCs depletion (33). Some chemotherapeutics such as gemcitabine, 5-fluorouracil, paclitaxel, and doxorubicin were shown to selectively eliminate MDSCs in the spleen, blood, and tumor beds in several mouse tumor models resulting in the enhancement of the function of immune effector cells (34–38). These findings reinforce the concept that depleting MDSCs has great therapeutic promise. In cancer patients, “conventional” therapies including surgical resection (39), radiotherapy (40) or chemotherapy with gemcitabine or 5-fluorouracil, showed a decrease of MDSCs leading to the immune recovery and tumor regression (35, 36). However, MDSC numbers and/or function have been assessed in few chemotherapy clinical trials and have shown mixed results.

Intensive investigations have been performed to reduce MDSCs trafficking to peripheral lymph nodes and tumor sites. Chemokine receptors are a key driving force for the migration of MDSCs and blocking the interactions with their ligands is a rational approach to inhibit MDSCs accumulation in the TME (41). In particular, therapeutic blockade of CCL2-CCR2 interaction by using CCL2 neutralizing antibodies or CCR2 antagonist has demonstrated promising antitumor efficacies in several preclinical cancer models (42–44). However, in a phase II clinical trial, was reported that carlumab (anti-CCL2 mAb) in patients with metastatic castration-resistant prostate cancer, induced a rapid rebound of the circulating concentration of free CCL2 to value higher than the pretreatment serum levels (45). The CCR5–CCL5 axis has also a critical role in tumor progression since it supports tumor invasion and migration of MDSCs to the tumor site (46). Indeed, by targeting the CCR5-CCL5 interaction, tumor growth and invasiveness were suppressed in colorectal, prostate, breast cancer and melanoma (47–50). Another well-characterized target to reduce MDSCs trafficking is the colony-stimulating factor 1 receptor (CSF1R) whose expression is restricted to monocytes and macrophages. Various inhibitors against CSF1R (such as IMC-CS4, GW2580, PLX3397, AMG820, and emactuzumab) have shown promising antitumor efficacies by inhibiting the survival of M-MDSCs and tumor associated macrophages (TAMs) and are being tested in combination with chemotherapy or immunotherapies in cancer patients (51). The following MDSCs inhibitors have been evaluated in clinical trials (52): Reparixin and AZD5069 (CXCR2 antagonists), respectively, in phase II for breast cancer and in phase Ib/II for advanced solid tumors and metastatic squamous cell carcinoma; Plexidartinib (CSF-1R inhibitor) in phase II for recurrent glioblastoma; Maraviroc (CCR5 antagonist) in phase I for metastatic colorectal cancer. The expansion and recruitment of MDSCs to the tumor sites is also mediated by MMP9. It has been shown that administration of amino-bisphosphonates drugs can prevent MMPs from undergoing prenylation, a post-translational modification that is essential for their function. As a result of reduced MMP9 prenylation, cleavage of the tyrosine kinase c-Kit is diminished, causing reduced mobilization of MDSCs (53). Amino-bisphosphonates have shown a good safety and tolerance and seem to exert therapeutic effects, making them promising candidates to target MDSCs (54–56). The inhibition of VEGF receptor signaling also leads to a reduction of MDSCs infiltration (57). Indeed, the tyrosine kinase inhibitor (TKI) sunitinib was reported to decrease the number of circulating MDSCs in renal cell carcinoma patients via blockade of VEGF and c-KIT signaling (58). Interestingly, sunitinib treatment resulted also in a significant reduction of STAT3 activation and ARG1 expression in M-MDSCs that was accompanied with an elevated activity and proliferation of CD8^+^ T cells (59).

Blockade of MDSCs immunosuppressive mechanisms represents the major therapeutic approach to re-establishing T-cells activity and immunotherapy success. MDSCs can be functionally inactivated by targeting their suppressive machinery. For example, disruption of the COX-2/prostaglandin E2 (PGE2) signaling has been successful in repressing MDSC-associated suppressive factors such as ARG1 expression and ROS production, and improving T-cells frequency and immune response (60, 61). Phosphodiesterase-5 (PDE-5) inhibitors are also able to inhibit MDSCs functions by the downregulation of iNOS and ARG1 expression and activities. In preclinical mouse models, administration of PDE-5 inhibitors, such as sildenafil and tadalafil, has been demonstrated to reactivate antitumor immunity through T-cells and natural killer (NK) cells and to prolong survival of tumor-bearing mice (62–64). Recent clinical trials with PDE-5 inhibitors have also shown enhanced intra-tumor T-cells activity and improved patients' outcome in head and neck squamous cell carcinoma (HNSCC) and metastatic melanoma (65–67). Blocking the immunosuppressive function of MDSCs can also be achieved by targeting phosphatidylinositol 3-kinase (PI3K). Knockout of PI3K was found to reduce the accumulation of G-MDSCs in tumor-bearing mice, breaking immune tolerance to cancer (68). Anti-inflammatory triterpenoids, have been demonstrate to reduce intracellular ROS production by MDSCs by upregulating the nuclear factor erythroid 2-related factor 2 (Nrf2) which plays an important role in the cellular protection against free radical damage (69). Moreover, synthetic triterpenoids, such as CCDO-IM and CCDO-Me, have shown promising anticancer results in phase I clinical trials (69, 70). Administration of ATRA, a vitamin A derivative binding to the retinoid receptor, also led to the downregulation of ROS production in MDSCs by activating the extracellular-signal regulated kinase (ERK)1/2 pathway (71). The selective class I histone deacetylase (HDAC) inhibitor entinostat has been reported to have an inhibitory effect on MDSCs immunosuppressive functions in several preclinical tumor models (72–74). Indeed, analysis of MDSCs response to entinostat revealed significantly reduced ARG1, iNOS, and COX-2 levels in both M- and G-MDSCs subsets. Interestingly, the combination of entinostat with immune checkpoint inhibitors resulted in prolonged survival and delayed tumor growth along with an increase of CD8^+^ T effector cells in tumor-bearing mice (73, 74). Clinical trials involving entinostat are currently underway (52). Recently, the inactivation of class II HDAC (HDAC6) with ricolinostat was found to further increase the inhibitory effect of entinostat on the MDSCs suppressive activity and on tumor progression (75). STAT3 is another promising target to reduce MDSCs immunosuppressive functions. Various approaches for STAT3 inhibition, such as inhibiting the (1) SH2 domain or dimerization, (2) upstream TKIs (e.g., JAK and Src inhibitors), (3) antisense oligonucleotides, and (4) peptide mimetics of physiological negative modulators of STAT3, have been tested in pre-clinical model and in clinical trials. However, their clinical use in advanced solid tumors have revealed limited efficacy or excessive toxicities (76). Recently, the antisense oligonucleotide STAT3 inhibitor, AZD9150, has been tested in phase I/Ib clinical trials for the treatment of diffuse large B-cell lymphoma. Systemic administration of AZD9150 in patients showed a positive immunomodulatory effect, with a marked decrease in G-MDSCs in the peripheral blood, and a meaningful antitumor activity. Trials to combine this agent with checkpoint-targeting immunotherapies are in progress (77).

Finally, another therapeutic approach used for targeting MDSCs is aimed to induce MDSCs differentiation, converting them into mature non-suppressive cells. One promising therapeutic appears to be ATRA which was reported to induce the rapid differentiation of MDSCs into mature myeloid cells, such as macrophages and DCs, and to improve T-cells response in cancer patients (78, 79). The mechanism of ATRA-induced differentiation of MDSCs involves specific up-regulation of glutathione synthase and neutralization of high ROS production in these cells (80). Several studies indicate that vitamin D3 is another agent that can promote myeloid cells maturation and reduce the number of MDSCs in cancer patient. In particular, vitamin D3 administration in HNSCC patients increased levels of IL-12, IFN-γ, and improved T-cells blastogenesis (81). Transcription factors instrumental for normal myeloid cells development, differentiation and function can also be a target to reducing aberrant myelopoiesis. In particular, the interferon regulatory factor (IRF)-8 is a “master regulator” of normal myelopoiesis, indispensable for producing monocytes, DCs and neutrophils from myeloid progenitors (82). Thus, enforced expression of IRF-8, either directly or indirectly, may facilitate myeloid differentiation and improves immunotherapy efficacy (83). Further, it has been hypothesized that tumor-induced IRF8 downregulation occurred through a STAT3-dependent interaction. Indeed, STAT3 inhibition can induce MDSCs differentiation into immunogenic DCs or macrophages (84, 85).

## MDSCs in Breast Cancer

Breast cancer (BC) is the most commonly occurring cancer and the leading cause of cancer-related deaths in females worldwide (86). Clinically, BC is a heterogeneous disease. Analyses of gene-expression profiling have identified three main groups of BC based on estrogenic receptor (ER), progesterone receptor (PR) and human epidermal growth factor receptor (HER2/neu) expression (87). This classification is critical for guiding treatments, which mainly include surgery (mastectomy or lumpectomy), radiotherapy, anthracycline-based chemotherapy or hormonal therapies with anti-HER-2 monoclonal antibodies (mAb), i.e., trastuzumab, pertuzumab, and TDM1 (88). Immunotherapy is not yet considered a routine form of treatment for BC patients. However, a recent pooled analysis of 1,954 breast tumor demonstrated that some BC, based on their different immunogenic sensitivity, can be distinguished into two discernible subtypes termed *immune benefit-enabled* and *immune benefit-disabled* which showed significant differences in distant metastasis-free survival (89). A better understanding of the factors that regulate BC immunogenicity will contribute to create more effective and personalized therapeutic strategies that target specific immunogenic subtypes. In particular, BC weak immunogenicity derive from mechanisms that diminish immune recognition and promote strong immunosuppression. Infiltration of immunosuppressive cells like T-regs, MDSCs or TAMs in the TME has been demonstrated to be the major mechanism of tumor escape from the immune system and the main cause in the reduction of the efficacy of immunotherapy (90). Indeed, circulating MDSCs in peripheral blood of BC patients have been shown to be elevated in all stages of the disease and to be positively correlated with clinical cancer stage and extensive metastatic tumor burden (91, 92). Conversely, tumors showing greater infiltration of about 50–60% of tumor-associated effector cells, such as cytotoxic T cells, memory T cells, NK cells, tend to be more immunogenic and more sensitive to chemotherapy. Thus, their presence has been associated with the suppression of metastatic recurrence resulting in a relatively good prognostic outcome (93–96). Most of the research on MDSCs in the TME has been performed in murine models, which have provided the first evidence that MDSCs are involved in the development and progression of BC. Thus, eliminating MDSCs can result in increased immune-mediated anti-tumor responses and decreased tumor-burden (97–101). Nevertheless, also in human it has been showed a direct correlation between MDSCs levels in the peripheral blood of BC patients, disease malignancy and poor prognosis. In one of the earliest study by Diaz-Montero et al. (91), the percentage and the absolute number of circulating MDSCs were significantly increased in cancer patients compared to normal volunteers. A population of MDSCs, defined as Lin^−/Lo^ HLA-DR^−^CD33^+^CD11b^+^, was detected in fresh whole blood from 106 BC patients. In these patients, it was found that both percentage and absolute number of circulating MDSCs were associated with the clinical cancer stage. Significant differences were observed in mean MDSCs between patients with early stages I/II cancer (1.96%) stage III (2.46%) and advanced stage IV (3.77%). Overall, stage IV patients with widely metastatic disease had the highest percent (4.37%). In that report, it has been also observed that MDSCs levels in the peripheral blood corresponded to circulating tumor cells levels, which are another emerging prognostic marker. Similarly, Solito et al. (102) also identified MDSCs (Lin^−/Lo^ HLA-DR^−^CD33^+^CD11b^+^) in 25 stage IV BC patients. They showed that subjects with higher circulating MDSCs > 3.17% (median) at baseline had a poorer overall survival (OS) than patients with circulating MDSCs ≤ 3.17%, with median OS times of 5.5 and 19.32 months, respectively. Interestingly, Yu et al. identified a unique population of MDSCs in BC with the phenotype CD45^+^CD33^+^CD13^+^CD14^−^CD15^−^. They found that these cells increased both in primary cancer tissues and in peripheral blood. The proportion of this cell population correlated with clinical stage and lymph node metastasis status in BC patients and exerted potent immunosuppressive activity on T cells. Further, they reported that IDO, a rate-limiting enzyme of tryptophan catabolism, was significantly upregulated in tumor-infiltrating MDSCs than in periphery, thereby suggesting a pivotal role in developing and maintaining MDSCs-mediated immunosuppressive functions in tissue (103). Recent studies also confirmed that tumor progression and invasion paralleled the development of MDSCs. For instance, Gonda et al. (104) reported that the levels of circulating MDSCs (CD33^+^CD11b^+^CD14^−^) in the peripheral blood were increased in BC patients compared with healthy controls. Moreover, MDSCs levels were considerably higher in preoperative patients and decreased in postoperative patients or following chemotherapy, while they reached again high levels in patients with recurrent disease. They found that, in preoperative patients, MDSCs levels positively correlated with IL-6 production while they negatively correlated with IFN-γ and IL-12 production. IL-12 is known to be a modulator of immune suppression which induces Th1 cells while IL-6 promotes a Th2-dominant status. Thus, the immune suppressive function of MDSCs in BC patients may involve multiple immunological pathways, which impair the Th cell balance promoting a shift from Th1 to Th2 predominance. Additionally, Bergenfelz et al. (92), reported an expansion of circulating CD14^+^HLA-DR^−/low^ M-MDSCs in patients with locoregional recurrence or metastatic BC, which was correlated with increased metastasis to lymph nodes and visceral organs, suggesting that circulating M-MDSCs could be a potential biomarker for disease progression and a guide to individualize efficient immunomodulatory treatments. Also Safarzadeh et al. (105) showed that M-MDSCs (HLA-DR^−^CD33^+^CD14^+^) represent a high percentage compared with the G-MDSCs (HLA-DR^−^CD33^+^CD15^+^) subpopulation in BC patients. A recent study found that cells with the M-MDSCs phenotype CD14^+^HLA-DR^−/low^ are present at significantly higher frequencies in early-stage BC patients (40 patients with clinical stages I/II), suggesting that M-MDSCs mostly participate to the development of BC by protecting tumor cells from immune attack. In particular, one of the suppressive mechanisms proposed by the authors for M-MDSCs-mediated immunosuppression is represented by ROS (106). Conversely, Toor et al. (107) found that BC patients had significant elevated levels of granulocytic CD33^+^ CD11b^+^HLA-DR^−/low^CD15^+^ MDSCs in the TME vs. surrounding healthy tissue whereas no significant differences were observed in their peripheral blood compared to healthy individuals. However, a weakness of this study may be the small number of patients included (23 patients). In BC, after differentiation and recruitment, MDSCs suppress T cells via several pathways including the ARG1, ROS, RNS, and NO pathways (108). Indeed, nitration/nitrosylation of T cell receptors (TCRs) and CD8 molecules on the surface of T cells induces T cell tolerance (109). The JAK/STAT pathway is also important in regulating the various functions of MDSCs. Indeed, the transcription factor STAT-3 modulates the expression of target genes involved in various proinflammatory functions. Among them, STAT-3 promotes IDO expression. As mentioned before, IDO act as a major immune regulator inhibiting immune surveillance and promoting immune tolerance by suppressing TCR-mediated activation of T cells, as well as inducing amplification of T-regs (110, 111). Besides their canonical immunosuppressive functions, MDSCs have also direct effects on BC cells contributing to invasiveness and metastasis through the activation of the intracellular phosphatase and tensin homolog (PTEN)/Akt pathway. Upregulation of Akt in MDSCs results in increased expression of MMPs, including MMP2, MMP13, and MMP14, in BC cells which in turn promote invasion and metastasis (108). Moreover, MDSCs can act as osteoclast progenitors promoting BC metastasis to the bone. Through NO signaling and cross talk with BC cells, MDSCs can differentiate into osteoclasts in the bone microenvironment to exacerbate osteolysis in metastasizing BC which represent important issue for BC patients, causing high morbidity and mortality (98). In summary, these studies further strengthen the observations that MDSCs numbers increase in patients with BC as compared to healthy people, suggesting that targeting MDSCs may significantly improve the effect of immunotherapy protocols in patients with BC. In preclinical studies it has been demonstrates that CCR5 antagonists inhibited the metastatic potential of basal BC and reduced tumor growth (49). CSF-1R inhibition and CXCR2 antagonism has also been used in combination to reduce TAMs and G-MDSCs populations and improve anti PD-1 efficacy (51, 112). Further, the HDAC inhibitor, entinostat, in combination with the checkpoint inhibitors anti–PD-1 and anti–CTLA-4, led to a significant suppression of G-MDSCs in the TME and significantly improved tumor-free survival in HER2/neu transgenic BC mouse model (74). Combination of entinostat with nivolumab and ipilimumab is, currently, under evaluation in a phase I trial in patients with invasive and metastatic BC (NCT02453620). Other clinical studies aimed to investigate the effect of MDSCs inhibitors in combination with immunotherapy are ongoing.

## MDSCs in Colorectal Cancer

Colorectal cancer (CRC) is the third most common cancer and the second cause of cancer deaths worldwide (86). Only 5–6% of CRC cases involve inherited genetic alterations while environmental factors, lifestyle (such as physical inactivity, smoking, alcohol consumption and obesity) and gut microbiota are responsible of ~90% of CRC occurrence (113). The current approaches to treat metastatic CRC (mCRC) involve multimodal therapy based on chemotherapy (including the combination of cytotoxic drugs) or targeted agents (such as bevacizumab, cetuximab, and panitumumab) (114). In the last few years, immunotherapy, which typically rely on the activation of T cells in the TME, has been considered for mCRC patients (115). Checkpoint inhibitors such as antibodies directed against cytotoxic T lymphocyte antigen-4 (CTLA-4) and programmed cell death protein (PD-1)/PD-1 ligand (PD-L1) resulted ineffective to produce durable clinical responses due to tumor-mediated immune evasion and resistance, caused by the presence, into the TME, of immunosuppressive cells like MDSCs (116). In CRC, MDSCs are widely considered the link between chronic inflammation and cancer. Indeed, patients with inflammatory bowel disease, such as ulcerative colitis, show an increased risk of developing CRC (117). Evidences from studies in mouse models of colitis-associated cancer (CAC) indicate that chronic inflammation can drive tumor initiation and progression by enhancing MDSCs accumulation and immune suppression (118, 119). Accumulating data also support a role for the microbiota in CRC carcinogenesis (120). Recent studies have shown that symbiotic bacteria like *Fusobacterium nucleatum* and *Helicobacter hepaticus* can exacerbate the development of cancer by inducing MDSCs expansion in the inflamed colon of mice (121, 122). Although both MDSCs subtypes have been found increased in several colon cancer mouse models, the expansion of G-MDSCs resulted much greater compared to M-MDSCs (118, 119, 122, 123). In CRC patients, at first, MDSCs were identified generally as CD33^+^HLA-DR^−^ (124, 125). Both circulating and tumor-infiltrating MDSCs have been found significantly expanded in patients with various stage of CRC compared with healthy donors. Interestingly, their frequencies were shown to increase with tumor stage and with the presence of nodal and/or distant metastasis, indicating a correlation with clinical cancer stage. These MDSCs displayed characteristics of immature myeloid cells expressing no level of the lineage markers CD3, CD14, CD19, and CD56. Notably, they showed up-regulation of CD18/CD11b expression, which is critical for cell adhesion and migration, suggesting the involvement of MDSCs in CRC tumor development (124). Further, Zhang et al. (125) demonstrated that Lin^−/low^HLA-DR^−^CD33^+^CD11b^+^ MDSCs had immunosuppressive effect on T cells and expressed high level of the ectonucleotidase molecule CD39, which plays a key role in mediating the suppressive activity of MDSCs on T cells, by converting immunostimulatory ATP into immunosuppressive adenosine. A better phenotypical characterization of MDSCs in CRC patients was originally reported by OuYang et al. (126). They observed an increased proportion of CD33^+^CD11b^+^HLA-DR^−^ MDSCs in peripheral blood and tumor tissues which correlated with advanced disease stages and tumor lymph node metastases. In particular, this population consisted for the major part of a M-MDSCs subset (CD33^+^CD11b^+^HLA-DR^−^CD14^+^CD15^−^) and an atypical G-MDSCs subset, with a moderate expression of the granulocyte-monocyte progenitor cell markers CD117 and a weak expression of the granulocytic marker CD15. These MDSCs populations were found to suppress both CD8^+^ and CD4^+^ T cells proliferation through the oxidative metabolism, including the generation of NO and ROS, as demonstrated by the high expression levels of the immune mediators ARG1, iNOS, and NOX2. Conversely, Toor et al. (127) identified CD33^+^CD11b^+^HLA-DR^−/low^CD15^+^ G-MDSCs as key players among others in CRC progression. They found a significant expansion of G-MDSCs in both circulation and in tumor tissues of 21 CRC patients with different tumor stages. In particular, circulating G-MDSCs were significantly elevated in CRC patients with regional and distant metastases and exerted their immunosuppressive functions trough the activation of ARG1. Several factors have been implicated in the regulation of the accumulation and the suppressive functions of MDSCs in CRC. IL-17 appears one of the main driving chemoattractant forces, especially for G-MDSCs, within the TME (128). In murine tumor models, IL-17 promotes MDSCs tumor infiltration, in a CXCL5/CXCR2-dependent manner, and enhances the immunosuppressive activity of MDSCs (129). Chun et al. (119) postulated that CCL2 acts as a neoplastic regulator of MDSCs, contributing to their intratumoral accumulation and to G-MDSC-mediated suppression of CD4^+^ and CD8^+^ T cells via STAT3-mediated pathway. Indeed, increased CCL2 in patients with early-stage colon cancer (colitis-associated CRC, adenocarcinomas, and adenomas) influences carcinogenesis inducing MDSCs. Thus, CCL2 neutralization may afford therapeutic opportunities to decreased MDSC accumulation and function. Recent data indicate that Yes-associated protein 1 (YAP1) and PTEN can mediate CRC tumorigenesis through the induction of MDSCs in the TME. In fact, Yang et al., describe that up-regulation of YAP1 in the tumor promoted MDSCs expansion through suppressing PTEN expression and subsequently inducing the secretion of GM-CSF (130). Further, inhibition of Kit has been demonstrated to enhance the antitumor activity of immune checkpoint inhibitors (anti–CTLA-4 and anti–PD-1) by selectively reducing the immunosuppressive M-MDSCs population in Colon26 mouse tumor model (131). The humanized anti-Kit mAb KTN0158 has also been evaluated in clinical trials for patients with Kit positive advanced solid tumors (NCT02642016). Notably, the inhibition of STAT3 signaling pathway with nifuroxazide inhibited lung and abdomen metastasis in mice and reduced the number of MDSCs in the blood, spleens and tumors, accompanied by the increased infiltration of CD8^+^ T cells (132). Targeting TRAIL-R2 with the agonist antibody DS-8273a was applied in a phase I clinical trial in patients with advanced cancers, including CRC patients, in combination with nivolumab and caused selective depletion of MDSCs without affecting mature myeloid or lymphoid cells (133). Thus, a better identification of the molecular mechanism driving MDSCs expansion in CRC may guide the future development of new therapeutic strategies for CRC patients based on targeting MDSCs.

## MDSCs in Melanoma

Melanoma is the most aggressive and fatal form of skin cancer with a high mortality rate. Primary melanoma is usually curable with surgery when diagnosticated in early stages (134). Nonetheless, melanoma is characterized by a lively progression that is correlated to rapid metastasis development to regional lymph nodes and distant organs as well as therapy resistance by reducing the patients median survival to <1 year (135). In fact, despite the recent introduction of encouraging immunotherapies such as ipilimumab and pembrolizumab, that target CTLA-4 and PD-1 respectively, the majority of patients experience resistance and tumor progression (136). This critical condition is partially due to the immunosuppressive mechanisms established within the TME mediated by immunoregulatory cells including T-regs and MDSCs that contributes to immune evasion (137). In particular, multiple reports have highlighted the role of MDSCs as one of the most important restrictions preventing efficient melanoma treatment (116). Several reports indicated an increased frequency of both M-MDSCs and G-MDSCs in melanoma patients (138–141). For instance, Jordan et al. demonstrated that the frequency of both M-MDSCs (Lin^−^CD11b^+^HLA-DR^−/low^CD33^+^CD14^+^) and G-MDSCs (Lin^−^CD11b^+^HLA-DR^−/low^CD33^+^CD14^−^) subsets was significantly increased in the peripheral blood of stage IV melanoma patients and was associated with disease progression and decreased OS (142). Similarly, Filipazzi et al. reported an expansion of CD14^+^CD11b^+^HLA-DR^−/low^ M-MDSCs in fresh whole blood from 70 advanced melanoma patients suggesting an inverse correlation with immune responses to cancer vaccine (138). Additionally, Weide et al. also reported that circulating CD14^+^CD11b^+^HLA-DR^low^ M-MDSCs were inversely correlated to both OS and the presence of functional antigen-specific T cells in patients with advanced melanoma (140). Conversely, more recently Stanojevic et al., demonstrated that HLA-DR^−/low^CD11b^+^CD33^low^Lin^−^CD14^−^CD15^+^ G-MDSCs population was significantly higher in different clinical melanoma stages according to both TNM and AJCC classification (143). Thus, MDSCs abrogation and inhibition, could be the next biggest aims for melanoma treatment (144). In fact, in the last few years, various preclinical studies have been focused in measuring and targeting MDSCs in melanoma patients, resulting in tumor growth inhibition and survival prolongation (145). Nevertheless, there are different ongoing clinical trials focused on evaluating the effect of new molecules that target MDSCs in melanoma patients such as ATRA), SX-682 or omaveloxolone in combination with classical immune checkpoint inhibitors (116, 144). ATRA, that has previously demonstrated to induce differentiation of MDSCs into macrophages and DCs in mice, (80) has been applied in a phase II clinical trial in combination with ipilimumab in melanoma patients. The study demonstrated that this combination improved the clinical outcome by increasing tumor antigen-specific T cell responses and reducing MDSCs frequency as compared to ipilimumab alone (146). SX-682 is a selective and potent antagonist of CXCR1/2 chemokine receptors that are expressed on both melanoma cells and MDSCs supporting tumor growth, immunosuppression and angiogenesis in response to CXCL1, CXCL2, or CXCL8 (147–149). Omaveloxolone (also referred as RTA408), is a semisynthetic oleanane triterpenoid that represses ROS production and NO signaling in MDSCs showing promising preclinical antitumor activity (150). Both SX-682 and RTA408 have been applied in two different clinical trials in combination, respectively with pembrolizumab (NCT03161431) and ipilimumab or nivolumab (NCT02259231) (116, 144, 151). Interestingly, MDSCs enrichment in melanoma patients has been frequently associated to heightened amounts of inflammatory mediators such as IFN-γ, IL-1β, IL-4, IL-13, TNF-α, toll-like receptor (TLR) ligands, and PGE2 that support MDSCs accumulation and activation (152, 153). PGE2 is one of the best-characterized prostaglandins synthesized by COX-2. Recently, we and others reported that COX-2 has a crucial role in melanoma development and progression by affecting patients progression free survival (PFS) (154–156). In particular, PGE2 production by MDSCs has been associated to ARG1 overexpression, STAT3 and STAT1 phosphorylation and IL-10, ROS, and NO production that are correlated to MDSCs suppressive activity (157–160). Thus, PGE2–dependent activation of MDSCs result to be a potent additional mechanism of tumor immune escape which is driven by COX-2 (161). Indeed, COX-2 pharmacologic inhibition reverts MDSCs suppressive phenotype by reducing the production of ROS and NO, the expression of ARG1 and restoring the differentiation of bone marrow cells (162, 163). Nevertheless, a better understanding is necessary to figure out which mechanisms PGE2 exploits for triggering MDSCs immunosuppressive effects in malignant melanoma. Recently, a new class of compound defined as hydrogen sulfide donors, has been shown to inhibit both the expansion and the suppressive functions of MDSCs in melanoma-bearing mice (164). Interesting results have also been achieved in the field of microRNAs (miRNAs) (165). miRNAs are relevant multifunctional post-transcriptional modulators of gene expression which have been reported to play a key-role in various human cancers including melanoma (166–171). Different evidences established an emerging role for miRNAs in the expansion and functional activation of MDSCs during tumor development (165). For instance, miR-155 has been shown to promote tumor growth by triggering MDSCs ripening, endurance and function through SOCS1 inhibition (172). More recently, Huber et al., discovered a set of miRNAs that are associated with the phenotypic and functional features of MDSCs in melanoma patients (173). Most importantly, they reported that higher expression of these miRNAs is correlated to shorter PFS in patients receiving ipilimumab and nivolumab (173). Finally, miRNAs identification as MDSC regulators, could be an additional and promising strategy to fight and monitor systemic immunosuppression that occur in melanoma patients, mainly driven by MDSCs.

## MDSCs in Prostate Cancer

Prostate cancer is the most commonly diagnosed cancer in males in the world and is responsible for about 20% of cancer-related deaths (174). Prostate cancer diagnosis is divided in low, intermediate and high risk according to Gleason patterns, prostate specific antigen (PSA) levels and clinical stage (175). Surgical or chemical androgen deprivation therapy (ADT) is the first-line treatment once the disease spreads outside the prostate in order to reduce circulating testosterone levels (176, 177). Nevertheless, an important percentage of patients experience resistance and tumor progresses to a more aggressive form referred as castration-resistant prostate cancer (CRPC) after 18–36 months (178, 179). This advanced form of prostate cancer is usually treated with classical chemotherapy regimens including docetaxel and cabazitaxel (179). Moreover, there are also novel hormone therapies available for CRPC such as abiraterone and enzalutamide (180, 181). In 2010, the U.S. Food and Drug Administration (FDA) approved PROVENGE (sipuleucel-T), the first immunotherapy agent for the treatment of patients with asymptomatic or minimally symptomatic metastatic CRPC. Sipuleucel-T stimulates T-cell immune response against prostate cancer cells by targeting prostatic acid phosphatase (PAP), an antigen that is highly expressed in most prostate cancer cells (182). Despite these recent advances, treatments only provide scanty survival benefits and most patients develop disease relapse (183). Investigating on the mechanisms that may drive prostate cancer progression, different data reported that it is surrounded by a complex TME (184, 185). In particular, MDSCs are the most renowned immune cells subset that has been reported to infiltrate the prostate TME (186–188). In fact, by evaluating the frequency of MDSCs in the blood of prostate cancer patients the CD14^+^HLA-DR^low^ monocytic subset result to be augmented compared with sex- and age-matched healthy donors, whereas it is decreased after ADT (39, 189). Conversely, Chi et al., reported that circulating CD33^+^CD11b^+^HLA-DR^−^CD14^−^ granulocytic MDSCs represented the major subtype of MDSCs in patients with prostate cancer and their level were significantly elevated compared with both healthy donors and patients with benign prostatic hyperplasia (BPH) (190). Interestingly, Idorn et al., showed that the levels of CD14^+^HLA-DR^low/−^ M-MDSCs were increased in both untreated and docetaxel-treated CRPC patients and that they were correlated with a shorter median OS, suggesting that MDSCs support prostate cancer progression (191). Additionally, they also reported a significant positive correlation between MDSCs and T-regs frequency in peripheral blood of CRPC patients denoting a cross-talk between these two immunomodulatory cells (191). This intricate scenario is orchestrated by different mediators such as cytokines, chemokines and growth factors that contribute to the accumulation of MDSCs in prostate tumors (192). In particular, elevated levels of IL-6 pro-inflammatory cytokine, have been reported to promote cancer cell growth and significantly correlate with MDSCs expansion (193–195). In fact, it has been showed, in mice, that high serum levels of IL-6 were positively associated to MDSCs recruitment (195). This data has been further reinforced by using IL-6 KO mice in which the inhibition of tumor-produced IL-6 significantly reduced MDSCs recruitment (195). Similarly, Chi et al. reported that MDSCs frequency was correlate with serum levels of IL-6 and IL-8 in prostate cancer patients (190). This IL-6-mediated immunosuppressive effect involves different signaling pathways including PI3K/PTEN/AKT pathway which in turn triggers MDSCs recruitment (196, 197). Interestingly, more recently, Calcinotto et al., reported that IL-23 cytokine is another important MDSC-secreted factor that drives CRPC progression in both human and mice by sustaining the growth and the endurance of prostate cancer cells as well as the transcription of androgen dependent genes such as Nkx3-1, Pbsn, and Fkbp5 (186, 198). Moreover, co-administration of anti IL-23 antibody with enzalutamide, reverted resistance to castration in tumor-bearing mice by reducing tumor volume and proliferation (186). These findings demonstrated that MDSCs are the major players involved in prostate cancer progression and resistance. Thus, immunotherapies focused on the inhibition of either MDSCs recruitment or the inhibition of other mediators that sustain MDSCs immunosuppressive effect (e.g., IL-6 and IL-23) can be a promising therapeutic strategy for prostate cancer patients. Several clinical trials targeting MDSCs in prostate cancer are ongoing (197). One promising agent is tasquinimod, an oral second-generation quinoline-3-carboxamide derivative (199). Tasquinimod inhibits S100A9 protein that interacts with the receptor for advanced glycation end products (RAGE) and TLR4, triggering the inflammatory response (200). S100A9 is also involved in MDSCs recruitment in solid tumors sustaining tumor growth and metastasis development (201). A phase II clinical trial demonstrated that tasquinimod improved both PFS and OS in prostate cancer patients compared to placebo (202, 203). Nonetheless, in a phase III randomized controlled trial, tasquinimod significantly improved PFS but did not improve OS (204). However, larger controlled clinical trials are needed to confirm and validate tasquinimod as a standard agent for the treatment of CRPC.

## MDSCs in Hepatocellular Carcinoma

Hepatocellular carcinoma (HCC) is one of the leading causes of cancer-related death worldwide. Cirrhosis and liver inflammation are frequently associated with HCC, and inflammation is considered one of the main factors driving hepatocarcinogenesis (205). HCC is a highly chemotherapy-resistant tumor and the applicability of most cytotoxic drugs is severely limited by the underlying liver cirrhosis. Currently, sorafenib and lenvatinib, oral multi-TKIs with antiangiogenic activity, are the most widely used systemic therapeutic agents which have showed increase in median survival in patients with unresectable HCC, respectively of 12.3 and 13.6 months (206, 207). Recently, other oral multi-TKIs, regorafenib and cabozantinib, have been added as second line systemic therapeutic options in patients with disease progression on sorafenib (208, 209). In the last few years, the interest in immunotherapies for HCC has been growing giving great opportunities for treating HCC with newer and more sophisticated agents (210). In particular, encouraging results has been obtained with the anti PD-1 mAbs nivolumab and pembrolizumab, which exhibited an objective tumor response of about 20% in HCC patients who had been previously treated with sorafenib (207, 211). Optimizing this response is challenging, especially because of the immune environment on which HCC arises. Although ~25% of HCC show high or moderate levels of lymphocyte infiltration (TILs), within the TME (212), they often prove insufficient to control tumor growth because the expansion of immunosuppressor cells like MDSCs and Tregs (213). Indeed, there is a general consensus that various dysfunctions of the immune system contribute to HCC development and progression (214, 215). In the chronic inflammatory milieu present in the liver of HCC patients, myeloid cells infiltrating the tumor can acquire suppressive capability and contribute to immune escape of HCC cells. In the last decade, the clinical importance of MDSCs in HCC patients has been investigated. Several authors have reported elevated level of total MDSCs with the phenotype HLA-DR^−/low^CD11b^+^CD33^+^ in HCC patients compared with healthy controls (216–218). In other studies, MDSCs were identified as CD14^+^HLA-DR^−/low^, which are considered to be M-MDSCs. These M-MDSCs were found to be significantly elevated in the peripheral blood or tumor of HCC patients compared with chronic hepatitis patients and healthy controls. Moreover, the frequency of circulating MDSCs, both total and M-MDSCs, was significantly correlated with reduced OS and tumor progression (213, 219, 220). Later, Hetta et al. observed that HCV-HCC patients with advanced stage had higher percentage of total MDSCs and M-MDSCs in the peripheral blood compared with those with early-stage HCC and healthy control. The frequency of M-MDSCs subsets was positively correlated with liver related laboratory parameters, especially AFP and ALT, which reflects a hepatic insult whereas, was inversely related to the frequency of CD4^+^, CD8^+^ T, and CD19^+^ B cells. Moreover, patients with chronic liver disease had a significantly higher percentage of MDSCs suggesting that an increased level of MDSCs may contribute to the progression from chronic hepatitis to HCC (221). In a recent publication, an extensive study on 183 HCC patients showed the prognostic value of CD14^+^HLA-DR^−/low^ M-MDSCs for predicting early recurrence (within 2 years) in patients undergoing curative resection. In particular, the authors observed a significant positive correlation between the frequency of MDSCs and the systemic immune-inflammation index (SII), which is a powerful prognostic indicator of poor outcome in HCC patients after resection. Thus, HCC patients with high MDSCs level and high SII level had significantly shorter time to recurrence (TTR) and OS than those with low MDSC level and low SII level (219). However, due the limitations of this study, such as relatively small cohort size, short follow-up time, and data from a single study center, the prognostic significance of MDSCs requires further validation. Clinical studies of MDSCs in HCC have mainly focused on analyzing M-MDSCs. Recently, Nan et al. employed a novel marker, LOX-1, to analyze G-MDSCs in HCC patients and determined that LOX-1^+^CD15^+^ cells were significantly increased both in the peripheral blood and in tumor tissue of patients compared with healthy controls and were positively related to OS. Moreover, LOX-1^+^CD15^+^ MDSCs suppressed T-cell proliferation through the ROS and ARG1 pathway and reduced interferon IFN-γ production (222). Mechanistically, also M-MDSCs isolated from the peripheral blood of HCC patients have been proven to be immunosuppressive by inducing CD4^+^CD25^+^Foxp3^+^ regulatory T cells and inhibiting autologous NK cells, as well as they shown to have high ARG1 activity (213, 223). Nonetheless, Shen and colleagues, described an immature subset of Lin^−^ HLA-DR^−^CD33^+^ MDSCs in the peripheral blood of patients with primary HCC and their frequency was found to be positively correlated with tumor stage and splenomegaly. In the same way, the immature MDSCs were able to inhibit tumor-specific T-cell responses and IFN-γ secretion through a suppressive mechanism involving ARG1 and iNOS enzymes (224). Regarding the mechanism of MDSCs expansion, it was found that the serum levels of suppressive cytokines like IL-10 and IL-13 as well as of tumor-promoting factors like G-CSF, VEGF and MMP-13 were significantly increased in patients with high frequency of MDSCs (220, 224). Indeed, these cytokines, that trigger JAK-STAT signaling pathways are considered to be the main regulators of the activation of MDSCs, which leads to stimulation of myelopoiesis and inhibition of myeloid-cells differentiation (225).

Most published studies on human MDSCs in HCC patients have been done using blood samples. Thus, in order to better understand the complex immunobiology of MDSC in HCC, different murine HCC models have been employed: carcinogen-induced, spontaneous and transplantable HCC. Although all tumor bearing mice demonstrated elevated MDSCs level (identified as CD11b^+^Gr-1^+^ cells), subtle differences in frequency, location and function of MDSCs were found among the murine models (226). Pre-clinical models of HCC have been also used to evaluate the ability of sorafenib to modulate MDSCs. Several studies have reported that sorafenib could enhance the antitumor immunity by reducing MDSCs in tumor-bearing mice (226, 227). On the other hand, targeting MDSCs with anti-Ly6G or anti-IL-6 antibody significantly reduced the frequency of Ly6G^+^ MDSCs in orthotopic liver tumors improving the therapeutic effect of sorafenib (228). However, Chen et al. (229) demonstrated that sorafenib increased the intratumoral infiltration of Gr-1^+^ MDSCs through the SDF1α/CXCR4 pathway while reduced the accumulation of Gr-1^+^ myeloid cells in the surrounding fibrotic liver tissue. Differences in these studies might depend on the mouse liver cancer model, the sorafenib dose or the gating strategy used. Further, recent studies have investigated the role of MDSCs in the efficacy of checkpoint inhibitors in mouse HCC models. Chiu et al. found that targeting the enzyme, ectonucleoside triphosphate diphosphohydrolase 2 (ENTPD2), which support the maintenance of MDSCs, enhanced the efficacy of PD-1/CTLA-4 blockade (230). Likewise, depletion of the cell cycle-related kinase (CCRK) reduced tumor-infiltrating MDSCs and increased intratumor CD8^+^ T cells, thus enhancing the efficacy of PD-L1 inhibitor to eradicate HCC (217). In addition, an *in vitro* study demonstrated that combination of sorafenib with an anti-CTLA-4 mAb restored the proliferation of CD8^+^ lymphocytes co-cultured with MDSCs (231). Radiotherapy is commonly used as alternative approaches for HCC patients who may experience serious adverse effects to chemotherapeutics. Interestingly, a decrease in percentages of CD14^+^HLA-DR^low/−^ MDSCs was observed in patients who received curative radiofrequency ablation (220). Recently, it has been reported that hypofractionated irradiation with high dose per fraction reduced the level of circulating MDSCs in two HCC tumor-bearing mouse models and decreased the expression of MDSC-related stimulatory cytokines: IL-6, G-CSF and RANTES (232). Collectively, these preclinical studies not only confirmed the roles of MDSCs in tumor formation and progression but also indicated the importance to reduce MDSCs in order to improve the efficacy of therapeutic strategies in HCC. However, these results remain to be confirmed in cancer patients. In this regard, a recent phase I/Ib study (NCT01839604) tested the effect of danvatirsen (AZD9150), a STAT3 oligonucleotide inhibitor, in 39 patients with advanced/metastatic HCC. At the end of the study the results reported that only one patient had a partial response. A phase I/IIa clinical trial is evaluating the outcome of HCC patients, progressing under sorafenib, following the treatment with regorafenib, a multi-TIKs that targets angiogenic (VEGFR1–3, TIE2), stromal (PDGFR-β, FGFR), and oncogenic receptor tyrosine kinases (KIT, RET, and RAF) in combination with nivolumab (NCT04170556).

## MDSCs in Lung Cancer

Lung cancer is one of the most commonly diagnosed malignancies that is strongly correlated with cigarette smoking and is a leading cause of cancer-related death (233). Lung cancer is generally divided into two types: small cell lung cancer (SCLC) and non-small cell lung cancer (NSCLC). Both SCLC and NSCLC are treated with similar chemotherapeutic agents often in combination such as cyclophosphamide, doxorubicin, and vincristine (CAV) or cyclophosphamide, doxorubicin and etoposide (CDE) (234–236). In addition, different targeted antibodies and immunomodulators are currently used for the treatment of lung cancer (237, 238). However, a high percentage of patients do not respond or develop resistance to treatment promoting cancer progression (239, 240). MDSCs represent, together with Tregs as well as TAMs, the major immunosuppressive cells that make up the TME in lung cancer patients (241). For lung cancer, the main body of literature reports increases of monocytic CD33^+^CD11b^+^CD14^+^ MDSCs or granulocytic-like CD33^+^CD11b^+^CD14^−^ MDSCs (242–245). For instance, Feng et al., defined MDSCs as CD11b^+^CD14^+^ expressing high levels of the proinflammatory molecule S100A8/A9 whose expression was highly correlated with the ability to suppress T-cells proliferation (244). Recently, de Goeje et al., showed for the first time that the immunoglobulin-like transcript 3 (ILT3) represent a novel immunosuppressive molecule expressed by defined MDSCs subsets in lung cancer patients. In particular, ILT3 high expression on a specific subset of G-MDSCs, defined as CD11b^+^CD14^−^HLA-DR^−^CD33^+^CD15^+^ILT3^high^, was correlated with reduced survival into NSCLC patients (246). Interestingly, increased frequency of both M-MDSCs (HLA-DR^−/low^CD11b^+^CD14^+^CD15^−^) and G-MDSCs (HLA-DR^−/low^ CD11b^+^CD14^−^ CD15^+^) has been found not only in the peripheral blood of patients but also in the tumor lesions. Indeed, a strong elevation of both tumor-infiltrating MDSCs subsets compared with the circulating subsets has been showed, confirming that the tumor site is characterized by the strongest immunosuppression. In particular, the frequency of tumor infiltrating and circulating G-MDSCs correlated with tumor progression (247). Among the different mediators that have been reported to regulate MDSCs suppressive functions, gp91phox, which is correlated to NADPH oxidase enzyme (248), results to be upregulated in MDSCs of lung cancer patients (242). The activity of NADPH oxidase enzyme translates into an increase in ROS production which mediates tumor immunosuppression and might thus represent a potential target for therapeutic intervention. Other important mediators involved in cancer immunosuppression are IDO and the adenosine (ADO)-producing enzymes CD39 and CD73 (249–253). It has been reported that ADO-producing enzymes are expressed in MDSCs isolated from the peripheral blood of NSCLC patients and favor their immunosuppressive function. Further analysis identified a novel MDSCs subpopulation enriched in CD39 and CD73 in tumor lesions of NSCLC patients defined as Lin^−^CD14^−^CD11b^+^CD39^+^CD73^+^ and Lin^−^CD14^+^CD11b^+^CD39^+^CD73^+^ that were found to be positively correlated to disease progression and were reduced after chemotherapy cycles suggesting them as predictive tools for chemotherapy response (254). Moreover, the ratio between Treg cells and G-MDSCs may also have an impact on the response to nivolumab treatment, since patients with a high frequency of circulating Tregs and low frequency of G-MDSCs show improved PFS in NSCLC patients (255). However, more research is needed to better understand the correlation between MDSCs and Tregs in this type of cancer. Given these evidences about the association between MDSCs and anticancer therapies, strategies focusing on the functional targeting of MDSCs are fast approaching clinical realization. For example, depletion of MDSCs increases the frequency and activity of NK and T cell effectors in the tumor and enhance therapeutic vaccination responses (256). Furthermore, it has been also demonstrated that dopamine receptor D2 (DR2) agonists and histamine type-2 receptor antagonists, such as carbegoline and cimetidine respectively, inhibit the progression of lung cancer in both human and mouse models by affecting at least in part MDSCs proliferation and function (30, 257). Interestingly, different natural compounds, such as resveratrol and curcumin, have been defined as novel synergistic agents for tumor immunotherapy. It has been demonstrated that resveratrol reduces *in vivo* lung cancer development and progression by inducing MDSCs apoptosis and reducing the recruitment of G-MDSCs (258). Likewise, curcumin reduced the frequency of MDSCs in the tumor and the spleen of tumor-bearing mice that was correlated to the reduction of IL-6 which is known to influence the function of MDSCs (259, 260). Giving the promising data regarding the targeting of MDSCs in mouse lung cancer, several clinical trials are now ongoing in NSCLC patients (NCT02922764; NCT03846310; NCT03801304; NCT04262388).

## Conclusion

To overcome tumor immune evasion is the new challenge of our era. Cancer immunotherapy has experienced remarkable advances in recent years, and significant improvements have been achieved in the treatment of several solid cancer types (e.g., melanoma, non-small cell lung cancer, bladder cancer). However, for most patients a favorable initial response to treatment changes afterwards, thereby leading to cancer relapse and recurrence. A key factor underlying the limited response to immunotherapies is the existence of multiple mechanisms mediating tumor immune suppression (261). In this context, MDSCs have been recognized to have a crucial role. Recent studies demonstrated the value of MDSCs in predicting the response to cancer immunotherapies. In particular, a close association of MDSCs level with patient response to the checkpoint inhibitors anti-CTLA4 (262, 263) and anti-PD-1 (264) has been observed. Moreover, a growing number of studies have demonstrated a significant correlation between circulating MDSCs frequency in cancer patients with tumor stage, metastatic spreading, and course of the disease. Indeed, a recent meta-analyses including 40 studies and 2,721 patients with solid cancer support the existence of an association between higher MDSCs levels and worse OS as well as shorter disease-free survival/progression-free survival/recurrence-free survival. The negative prognostic value of MDSCs was observed for all MDSCs subtypes, most tumor types, and all tumor stages suggesting a potential novel and promising use of MDSCs as prognostic biomarkers and/or therapeutic target (265). Initial studies monitored MDSCs in cancer patients, analyzed total MDSCs population (G-and M-MDSC together). The diversity of cell surface markers used to identify the main subsets of tumor-derived MDSCs in human is very high, which is in part due to the differences in the factors that are involved in the development and activation of MDSCs. The complexity of the human MDSCs phenotype is summarized in Table 1, with the main MDSCs phenotypes expanded in cancer patients and the common immunosuppressive mechanisms. The M-MDSCs subset defined as HLA-DR^−/low^CD14^+^, resulted to be predominant in melanoma, breast cancer and hepatocellular carcinoma. Conversely, in colorectal cancer G-MDSCs defined as HLA-DR^−/low^ CD15^+^ were the most abundant in both circulation and in tumor tissues. In prostate cancer and in lung cancer both G-MDSCs and M-MDSCs subsets were significantly elevated in patients and positively correlated to disease progression. However, despite most of the suppressive mechanisms and phenotype differences reported seemed shared among MDSCs subsets and tumor types, it is necessary to further dissect their role in order to define whether these differences are real or related to some bias from analysis of some markers/mechanisms. Numerous preclinical studies carried out in mouse tumor models, have showed that targeting MDSCs improved the effect of anti-cancer therapies (266–268). Although tumor mouse models could be useful for a better understanding of the mechanisms of induction, expansion, trafficking, and function of MDSCs in tumor, and for a rapid screening of anti-MDSCs agents *in vivo*, the translation in human is not so straightforward. First, the identification of human MDSCs phenotype is still challenging, owing the great heterogeneity of MDSCs in different cancers. Second, most human studies focus only on circulating MDSCs while little is known about tumor infiltrating MDSCs. Thus, a better and univocal characterization of the predominant subsets of MDSCs in several types of cancer as well as their further evaluation at the tumor site represent a compelling requirement in order to develop new effective strategies for targeting MDSCs. It is well-known that different subsets of MDSCs could use different mechanisms to suppress T-cells function. Therefore, the identification of the specific immunosuppressive mechanism is also essential to find the proper agent to block it and, consequently, to inhibit their function. Reduction of MDSCs expansion and recruitment to peripheral lymph nodes and tumor sites, inhibition of MDSC's suppressive activity and promotion of their differentiation into mature non-suppressive cells are the current therapeutic approaches that are being investigated to target MDSCs (Figure 1). So far, only few agents approved by FDA have been reported to have direct effects on MDSCs accumulation, maturation, and function (e.g., ATRA, Vitamin D, Suitinib, Gemcitabine, Bevacizumab, Tadalafil). However, a wide number of therapies and combination therapies are currently being tested in human clinical trials (Table 2) demonstrating an improvement of the patients' clinical outcome (146, 202, 203). In sight of this, further studies are needed to identify or confirm key mechanisms and upstream signals involved in MDSCs generation, expansion and immunosuppressive function in different malignancies. Advances in this field should facilitate rational design of new strategies to target MDSCs in cancer in order to enhance clinical responses to current immunotherapies and improve OS in patients.

**Table 1:** Phenotype and immunosuppressive features of MDSCs subsets in cancer patients.

**MDSCs type**	**Phenotype**	**Immunosuppressive features**	**Tumor**	**Site**	**References**
T-MDSCs	Lin^−/Lo^ HLA-DR^−^ CD33^+^CD11b^+^	-	BC	PBMCs	(91, 102)
T-MDSCs	Lin^−/Lo^ HLA-DR^−^ CD33^+^CD11b^+^	CD39	CRC	PBMCs	(125)
T-MDSCs	Lin^−/Lo^ HLA-DR^−^ CD33^+^	ARG1, iNOS, MMP-13, VEGF	HCC	PBMCs	(224)
T-MDSCs	CD45^+^CD11b^+^ CD33^+^	-	CRC	TT	(125)
T-MDSCs	HLA-DR^−^ CD33^+^	-	CRC	PBMCs/TT	(124)
T-MDSCs	CD33^+^CD45^+^CD13^+^CD14^−^ CD15^−^	IDO, IL-4R	BC	PBMCs/TT	(103)
T-MDSCs	CD33^+^CD11b^+^CD14^−^	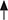 IL-6 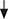 IL-12, INF-γ	BC	PBMCs	(104)
T-MDSCs	HLA-DR^−^ CD33^+^CD11b^+^	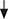 INF-γ	HCC	PBMCs/TT	(217, 218)
M-MDSCs	HLA-DR^−/low^CD14^+^	HMGB1, ARG1, S100P, MMP-9, MMP-25 ROS	BC	PBMCs	(92, 105, 106)
M-MDSCs	HLA-DR^−/low^CD14^+^	-	PC	PBMCs	(39, 191)
M-MDSCs	HLA-DR^−/low^CD14^+^	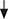 INF-γ 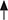 IL-10, IL-13, VEGF	HCC	PBMCs/TT	(213, 219, 220)
M-MDSCs	HLA-DR^−/low^CD14^+^	Nkp30 blocking	HCC	PBMCs/TT	(223)
M-MDSCs	HLA-DR^−/low^CD14^+^	gp91phox	NSCLC	PBMCs	(242)
M-MDSCs	CD33^+^CD11b^+^ HLA-DR^−^ CD14^+^CD15^−^	ARG1, CD39, iNOS, CXCR4	CRC	PBMCs/TT	(126)
M-MDSCs	CD33^+^CD11b^+^ HLA-DR^−/low^ CD14^+^	TGF-β	MEL	PBMCs	(140–142)
M-MDSCs	CD33^+^CD11b^+^ HLA-DR^−/low^ CD14^+^ CD15^−^	-	HCC	PBMCs/TT	(216)
M-MDSCs	CD33^+^CD11b^+^ HLA-DR^−^CD14^+^	-	HCC	PBMCs/TT	(221)
M-MDSCs	CD11b^+^CD14^+^S100A9^+^	ARG1, iNOS, IL-4Rα, IL-10	NSCLC	PBMCs	(224)
M-MDSCs	CD16^low^CD33^+^CD11b^+^ HLA-DR^−^ CD14^+^CD15^+^	ARG1, ROS	NSCLC	PBMCs	(245)
M-MDSCs	CD11b^+^ HLA-DR^−/low^ CD14^+^ CD15^−^	CCR5, PDL-1	NSCLC	TT	(247)
M-MDSCs	Lin^−^CD11b^+^ CD14^+^ CD73^+^ CD39^+^	IL-4R, HIF-1α, IL-10, COX-2	NSCLC	PBMCs/TT	(254)
G-MDSCs	HLA-DR^−/low^CD15^+^	-	BC	PBMCs	(105)
G-MDSCs	CD15^+^	ARG1	BC	TT	(107)
G-MDSCs	CD33^+^CD11b^+^ HLA-DR^−^ CD17^+^CD15^+^	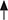 ROS; PDL-1	CRC	PBMCs/TT	(126)
G-MDSCs	CD33^+^CD11b^+^ HLA-DR^−/low^ CD15^+^	ARG1	CRC	PBMCs/TT	(127)
G-MDSCs	CD33^+^CD11b^+^ HLA-DR^−^ CD14^−^	-	MEL	PBMCs	(142)
G-MDSCs	CD33^low^CD11b^+^ HLA-DR^−/low^ CD14^−^CD15^+^	-	MEL	PBMCs	(143)
G-MDSCs	CD33^+^CD11b^+^ HLA-DR^−^ CD14^−^	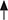 IL-6, IL-8	PC	PBMCs	(190)
G-MDSCs	CD33^+^CD11b^+^CD15^+^	IL-23	PC	TT	(186)
G-MDSCs	CD33^+^CD11b^+^ HLA-DR^−/low^ CD14^−^CD15^+^	-	HCC	PBMCs/TT	(216)
G-MDSCs	LOX-1^+^CD15^+^	ROS, ARG1	HCC	PBMCs/TT	(222)
G-MDSCs	CD33^+^CD11b^+^ CD14^−^CD15^+^	ARG1, iNOS, IL-4R, INF-γR	NSCLC	PBMCs	(243)
G-MDSCs	CD16^low^CD33^+^CD11b^+^ HLA-DR^−^ CD14^−^CD15^+^	ARG1, ROS	NSCLC	PBMCs	(245)
G-MDSCs	CD33^+^CD11b^+^ HLA-DR^−^ CD14^−^CD15^+^ILT3^high^	-	NSCLC	PBMCs	(246)
G-MDSCs	CD11b^+^ HLA-DR^−/low^ CD14^−^ CD15^+^	CCR5, PDL-1	NSCLC	TT	(247)
G-MDSCs	Lin^−^CD11b^+^ CD14^−^ CD73^+^ CD39^+^	IL-4R, HIF-1α, IL-10, COX-2	NSCLC	PBMCs/TT	(254)

*BC, breast cancer; CRC, colorectal cancer; HCC, hepatocellular carcinoma; MEL, melanoma; NSCLC, non-small cell lung cancer; PBMCs, peripheral blood mononuclear cells; PC, prostate cancer; TT, tumor tissue; M-MDSCs, monocytic-MDSCs; G-MDSCs, granulocytic-MDSCs; T-MDSCs, total-MDSCs*.

**Table 2:** Summary of clinical trials targeting MDSCs in cancer patients.

**Drug**	**Target**	**Combination partner**	**Tumor**	**ClinicalTrials.gov identifier**
ENTINOSTAT	class I HDAC	Nivolumab	BC	NCT02453620
IPI-549	PI3K	Nivolumab	NSCLC, MEL, BC	NCT02637531
IPI-549	PI3K	Tecentriq and Abraxane	BC	NCT03961698
REPARIXIN	CXCR2	Paclitaxel	BC	NCT02370238
AB928	A_2a_R and A_2b_R	IPI-549, PLD, NP	BC	NCT03719326
DS-8273a	TRAIL-R2	Nivolumab	CRC	NCT02076451
PEXIDARTINIB	CSF1R	Durvalumab	CRC	NCT02777710
MARAVIROC	CCR5	-	CRC	NCT01349036
DANVATIRSEN (AZD9150)	STAT3	-	HCC	NCT01839604
REGORAFENIB	multi-TKIs	Nivolumab	HCC	NCT04170556
ATRA	Retinoic acid receptor	Ipilimumab	MEL	NCT02403778
SX682	CXCR1/2	Pembrolizumab	MEL	NCT03161431
RTA408	Nrf-2	Ipilimumab/Nivolumab	MEL	NCT02259231
Tasquinimod	S100A9	-	PC	NCT01234311
AZD5069	CXCR2	Enzalutamide	PC	NCT03177187
Granocyte	G-CSF	Cabazitaxel plus Prednisone	PC	NCT02961257
RGX-104	LXR	Nivolumab/Ipilimumab/ Docetaxel/Pembrolizumab	NSCLC	NCT02922764
AB928	A_2a_R and A_2b_R	Carboplatin/Pemetrexed Pembrolizumab	NSCLC	NCT03846310
vinorelbine	Cytotoxic	Atezolizumab	NSCLC	NCT03801304
Oleclumab	CD73	Durvalumab	NSCLC	NCT04262388
PD-0360324	CSF1	Avelumab	NSCLC, MEL, BC	NCT02554812
ARRY-382	CSF1R	Pembrolizumab	NSCLC, MEL,	NCT02880371

*AR, adenosine receptor; BC, breast cancer; CCR5, C-C chemokine receptor type 5; CXCR1/2, C-X-C motif chemokine receptor 1/2; CRC, colorectal cancer; CSF1, colony-stimulating factor 1; CSF1R, colony-stimulating factor 1 receptor; G-CSF, granulocyte colony-stimulating factor; HCC, hepatocellular carcinoma; HDAC, histone deacetylase; LXR, liver X receptor; MEL, melanoma; NP, nanoparticle albumin-bound paclitaxel; Nrf-2, nuclear factor erythroid 2-related factor 2; NSCLC, non-small cell lung cancer; PC, prostate cancer; PI3K, phosphatidylinositol 3-kinase; PLD, pegylated liposomal doxorubicin; STAT3, signal transducer and activator of transcription-3; TKIs, tyrosine kinase inhibitors; TRAIL-R2, TNF-related apoptosis-induced ligand receptor 2*.

## Author Contributions

PD and GE did the bibliographic research and wrote the manuscript. AI critically revised the article for intellectual content. All authors contributed to the article and approved the submitted version.

## Conflict of Interest

The authors declare that the research was conducted in the absence of any commercial or financial relationships that could be construed as a potential conflict of interest.

## References

[1] Kim R Emi M Tanabe K. Cancer immunoediting from immune surveillance to immune escape. Immunology. (2007) 121:1–14. 10.1111/j.1365-2567.2007.02587.x 17386080 PMC2265921

[2] Gabrilovich DI Nagaraj S. Myeloid-derived suppressor cells as regulators of the immune system. Nat Rev Immunol. (2009) 9:162–74. 10.1038/nri2506 19197294 PMC2828349

[3] Tcyganov E Mastio J Chen E Gabrilovich DI. Plasticity of myeloid-derived suppressor cells in cancer. Curr Opin Immunol. (2018) 51:76–82. 10.1016/j.coi.2018.03.009 29547768 PMC5943174

[4] Millrud CR Bergenfelz C Leandersson K. On the origin of myeloid-derived suppressor cells. Oncotarget. (2017) 8:3649–65. 10.18632/oncotarget.12278 27690299 PMC5356220

[5] Bruns H Bottcher M Qorraj M Fabri M Jitschin S Dindorf J. CLL-cell-mediated MDSC induction by exosomal miR-155 transfer is disrupted by vitamin D. Leukemia. (2017) 31:985–8. 10.1038/leu.2016.378 28008175

[6] Messmer MN Netherby CS Banik D Abrams SI. Tumor-induced myeloid dysfunction and its implications for cancer immunotherapy. Cancer Immunol Immunother. (2015) 64:1–13. 10.1007/s00262-014-1639-3 25432147 PMC4282948

[7] Gabrilovich DI Velders MP Sotomayor EM Kast WM. Mechanism of immune dysfunction in cancer mediated by immature Gr-1+ myeloid cells. J Immunol. (2001) 166:5398–406. 10.4049/jimmunol.166.9.5398 11313376

[8] Kusmartsev S Su Z Heiser A Dannull J Eruslanov E Kubler H. Reversal of myeloid cell-mediated immunosuppression in patients with metastatic renal cell carcinoma. Clin Cancer Res. (2008) 14:8270–8. 10.1158/1078-0432.CCR-08-0165 19088044

[9] Lee JM Seo JH Kim YJ Kim YS Ko HJ Kang CY. The restoration of myeloid-derived suppressor cells as functional antigen-presenting cells by NKT cell help and all-trans-retinoic acid treatment. Int J Cancer. (2012) 131:741–51. 10.1002/ijc.26411 21898392

[10] Gabrilovich DI Ostrand-Rosenberg S Bronte V. Coordinated regulation of myeloid cells by tumours. Nat Rev Immunol. (2012) 12:253–68. 10.1038/nri3175 22437938 PMC3587148

[11] Bronte V Brandau S Chen SH Colombo MP Frey AB Greten TF. Recommendations for myeloid-derived suppressor cell nomenclature and characterization standards. Nat Commun. (2016) 7:12150. 10.1038/ncomms12150 27381735 PMC4935811

[12] Ostrand-Rosenberg S Fenselau C. Myeloid-derived suppressor cells: immune-suppressive cells that impair antitumor immunity and are sculpted by their environment. J Immunol. (2018) 200:422–31. 10.4049/jimmunol.1701019 29311384 PMC5765878

[13] Youn JI Nagaraj S Collazo M Gabrilovich DI. Subsets of myeloid-derived suppressor cells in tumor-bearing mice. J Immunol. (2008) 181:5791–802. 10.4049/jimmunol.181.8.5791 18832739 PMC2575748

[14] Movahedi K Guilliams M Van den Bossche J Van den Bergh R Gysemans C Beschin A. Identification of discrete tumor-induced myeloid-derived suppressor cell subpopulations with distinct T cell-suppressive activity. Blood. (2008) 111:4233–44. 10.1182/blood-2007-07-099226 18272812

[15] Condamine T Mastio J Gabrilovich DI. Transcriptional regulation of myeloid-derived suppressor cells. J Leukoc Biol. (2015) 98:913–22. 10.1189/jlb.4RI0515-204R 26337512 PMC4661041

[16] Groth C Hu X Weber R Fleming V Altevogt P Utikal J. Immunosuppression mediated by myeloid-derived suppressor cells (MDSCs) during tumour progression. Br J Cancer. (2019) 120:16–25. 10.1038/s41416-018-0333-1 30413826 PMC6325125

[17] Youn JI Gabrilovich DI. The biology of myeloid-derived suppressor cells: the blessing and the curse of morphological and functional heterogeneity. Eur J Immunol. (2010) 40:2969–75. 10.1002/eji.201040895 21061430 PMC3277452

[18] Lee CR Kwak Y Yang T Han JH Park SH Ye MB. Myeloid-derived suppressor cells are controlled by regulatory T cells via TGF-beta during murine colitis. Cell Rep. (2016) 17:3219–32. 10.1016/j.celrep.2016.11.062 28009291

[19] Fujimura T Ring S Umansky V Mahnke K Enk AH. Regulatory T cells stimulate B7-H1 expression in myeloid-derived suppressor cells in ret melanomas. J Invest Dermatol. (2012) 132:1239–46. 10.1038/jid.2011.416 22189788

[20] Motallebnezhad M Jadidi-Niaragh F Qamsari ES Bagheri S Gharibi T Yousefi M. The immunobiology of myeloid-derived suppressor cells in cancer. Tumour Biol. (2016) 37:1387–406. 10.1007/s13277-015-4477-9 26611648

[21] Youn JI Collazo M Shalova IN Biswas SK Gabrilovich DI. Characterization of the nature of granulocytic myeloid-derived suppressor cells in tumor-bearing mice. J Leukoc Biol. (2012) 91:167–81. 10.1189/jlb.0311177 21954284 PMC3250305

[22] Condamine T Dominguez GA Youn JI Kossenkov AV Mony S Alicea-Torres K. Lectin-type oxidized LDL receptor-1 distinguishes population of human polymorphonuclear myeloid-derived suppressor cells in cancer patients. Sci Immunol. (2016) 1:aaf8943. 10.1126/sciimmunol.aaf8943 28417112 PMC5391495

[23] Mandruzzato S Brandau S Britten CM Bronte V Damuzzo V Gouttefangeas C. Toward harmonized phenotyping of human myeloid-derived suppressor cells by flow cytometry: results from an interim study. Cancer Immunol Immunother. (2016) 65:161–9. 10.1007/s00262-015-1782-5 26728481 PMC4726716

[24] Gabrilovich DI. Myeloid-derived suppressor cells. Cancer Immunol Res. (2017) 5:3–8. 10.1158/2326-6066.CIR-16-0297 28052991 PMC5426480

[25] Khaled YS Ammori BJ Elkord E. Myeloid-derived suppressor cells in cancer: recent progress and prospects. Immunol Cell Biol. (2013) 91:493–502. 10.1038/icb.2013.29 23797066

[26] Solito S Marigo I Pinton L Damuzzo V Mandruzzato S Bronte V. Myeloid-derived suppressor cell heterogeneity in human cancers. Ann N Y Acad Sci. (2014) 1319:47–65. 10.1111/nyas.12469 24965257

[27] Stewart TJ Smyth MJ. Improving cancer immunotherapy by targeting tumor-induced immune suppression. Cancer Metastasis Rev. (2011) 30:125–40. 10.1007/s10555-011-9280-5 21249424

[28] Bronte V Serafini P Apolloni E Zanovello P. Tumor-induced immune dysfunctions caused by myeloid suppressor cells. J Immunother. (2001) 24:431–46. 10.1097/00002371-200111000-00001 11759067

[29] Qin H Lerman B Sakamaki I Wei G Cha SC Rao SS. Generation of a new therapeutic peptide that depletes myeloid-derived suppressor cells in tumor-bearing mice. Nat Med. (2014) 20:676–81. 10.1038/nm.3560 24859530 PMC4048321

[30] Zheng Y Xu M Li X Jia J Fan K Lai G. Cimetidine suppresses lung tumor growth in mice through proapoptosis of myeloid-derived suppressor cells. Mol Immunol. (2013) 54:74–83. 10.1016/j.molimm.2012.10.035 23220070

[31] Weiss JM Subleski JJ Back T Chen X Watkins SK Yagita H. Regulatory T cells and myeloid-derived suppressor cells in the tumor microenvironment undergo Fas-dependent cell death during IL-2/alphaCD40 therapy. J Immunol. (2014) 192:5821–9. 10.4049/jimmunol.1400404 24808361 PMC4048774

[32] Dang Y Rutnam ZJ Dietsch G Lu H Yang Y Hershberg R. TLR8 ligation induces apoptosis of monocytic myeloid-derived suppressor cells. J Leukoc Biol. (2018) 103:157–64. 10.1002/JLB.5AB0217-070R 29345064 PMC6721845

[33] Condamine T Kumar V Ramachandran IR Youn JI Celis E Finnberg N. ER stress regulates myeloid-derived suppressor cell fate through TRAIL-R-mediated apoptosis. J Clin Invest. (2014) 124:2626–39. 10.1172/JCI74056 24789911 PMC4038578

[34] Vincent J Mignot G Chalmin F Ladoire S Bruchard M Chevriaux A. 5-Fluorouracil selectively kills tumor-associated myeloid-derived suppressor cells resulting in enhanced T cell-dependent antitumor immunity. Cancer Res. (2010) 70:3052–61. 10.1158/0008-5472.CAN-09-3690 20388795

[35] Eriksson E Wenthe J Irenaeus S Loskog A Ullenhag G. Gemcitabine reduces MDSCs, tregs and TGFbeta-1 while restoring the teff/treg ratio in patients with pancreatic cancer. J Transl Med. (2016) 14:282. 10.1186/s12967-016-1037-z 27687804 PMC5041438

[36] Kanterman J Sade-Feldman M Biton M Ish-Shalom E Lasry A Goldshtein A. Adverse immunoregulatory effects of 5FU and CPT11 chemotherapy on myeloid-derived suppressor cells and colorectal cancer outcomes. Cancer Res. (2014) 74:6022–35. 10.1158/0008-5472.CAN-14-0657 25209187

[37] Alizadeh D Trad M Hanke NT Larmonier CB Janikashvili N Bonnotte B. Doxorubicin eliminates myeloid-derived suppressor cells and enhances the efficacy of adoptive T-cell transfer in breast cancer. Cancer Res. (2014) 74:104–18. 10.1158/0008-5472.CAN-13-1545 24197130 PMC3896092

[38] Sevko A Michels T Vrohlings M Umansky L Beckhove P Kato M. Antitumor effect of paclitaxel is mediated by inhibition of myeloid-derived suppressor cells and chronic inflammation in the spontaneous melanoma model. J Immunol. (2013) 190:2464–71. 10.4049/jimmunol.1202781 23359505 PMC3578135

[39] Brusa D Simone M Gontero P Spadi R Racca P Micari J. Circulating immunosuppressive cells of prostate cancer patients before and after radical prostatectomy: profile comparison. Int J Urol. (2013) 20:971–8. 10.1111/iju.12086 23421558

[40] Postow MA Callahan MK Barker CA Yamada Y Yuan J Kitano S. Immunologic correlates of the abscopal effect in a patient with melanoma. N Engl J Med. (2012) 366:925–31. 10.1056/NEJMoa1112824 22397654 PMC3345206

[41] Homey B Muller A Zlotnik A. Chemokines: agents for the immunotherapy of cancer? Nat Rev Immunol. (2002) 2:175–84. 10.1038/nri748 11913068

[42] Izhak L Wildbaum G Zohar Y Anunu R Klapper L Elkeles A. A novel recombinant fusion protein encoding a 20-amino acid residue of the third extracellular (E3) domain of CCR2 neutralizes the biological activity of CCL2. J Immunol. (2009) 183:732–9. 10.4049/jimmunol.0802746 19535619

[43] Li X Yao W Yuan Y Chen P Li B Li J. Targeting of tumour-infiltrating macrophages via CCL2/CCR2 signalling as a therapeutic strategy against hepatocellular carcinoma. Gut. (2017) 66:157–67. 10.1136/gutjnl-2015-310514 26452628

[44] Loberg RD Ying C Craig M Day LL Sargent E Neeley C. Targeting CCL2 with systemic delivery of neutralizing antibodies induces prostate cancer tumor regression *in vivo*. Cancer Res. (2007) 67:9417–24. 10.1158/0008-5472.CAN-07-1286 17909051

[45] Pienta KJ Machiels JP Schrijvers D Alekseev B Shkolnik M Crabb SJ. Phase 2 study of carlumab (CNTO 888), a human monoclonal antibody against CC-chemokine ligand 2 (CCL2), in metastatic castration-resistant prostate cancer. Invest New Drugs. (2013) 31:760–8. 10.1007/s10637-012-9869-8 22907596

[46] Umansky V Blattner C Gebhardt C Utikal J. CCR5 in recruitment and activation of myeloid-derived suppressor cells in melanoma. Cancer Immunol Immunother. (2017) 66:1015–23. 10.1007/s00262-017-1988-9 28382399 PMC11029643

[47] Robinson SC Scott KA Wilson JL Thompson RG Proudfoot AE Balkwill FR. A chemokine receptor antagonist inhibits experimental breast tumor growth. Cancer Res. (2003) 63:8360–5. 14678997

[48] Tan MC Goedegebuure PS Belt BA Flaherty B Sankpal N Gillanders WE. Disruption of CCR5-dependent homing of regulatory T cells inhibits tumor growth in a murine model of pancreatic cancer. J Immunol. (2009) 182:1746–55. 10.4049/jimmunol.182.3.1746 19155524 PMC3738070

[49] Velasco-Velazquez M Jiao X De La Fuente M Pestell TG Ertel A Lisanti MP. CCR5 antagonist blocks metastasis of basal breast cancer cells. Cancer Res. (2012) 72:3839–50. 10.1158/0008-5472.CAN-11-3917 22637726

[50] Blattner C Fleming V Weber R Himmelhan B Altevogt P Gebhardt C. CCR5^+^ myeloid-derived suppressor cells are enriched and activated in melanoma lesions. Cancer Res. (2018) 78:157–67. 10.1158/0008-5472.CAN-17-0348 29089297

[51] Cannarile MA Weisser M Jacob W Jegg AM Ries CH Ruttinger D. Colony-stimulating factor 1 receptor (CSF1R) inhibitors in cancer therapy. J Immunother Cancer. (2017) 5:53. 10.1186/s40425-017-0257-y 28716061 PMC5514481

[52] Law AMK Valdes-Mora F Gallego-Ortega D. Myeloid-derived suppressor cells as a therapeutic target for cancer. Cells. (2020) 9:561. 10.3390/cells9030561 32121014 PMC7140518

[53] Melani C Sangaletti S Barazzetta FM Werb Z Colombo MP. Amino-biphosphonate-mediated MMP-9 inhibition breaks the tumor-bone marrow axis responsible for myeloid-derived suppressor cell expansion and macrophage infiltration in tumor stroma. Cancer Res. (2007) 67:11438–46. 10.1158/0008-5472.CAN-07-1882 18056472 PMC2646404

[54] Porembka MR Mitchem JB Belt BA Hsieh CS Lee HM Herndon J. Pancreatic adenocarcinoma induces bone marrow mobilization of myeloid-derived suppressor cells which promote primary tumor growth. Cancer Immunol Immunother. (2012) 61:1373–85. 10.1007/s00262-011-1178-0 22215137 PMC3697836

[55] Sanford DE Porembka MR Panni RZ Mitchem JB Belt BA Plambeck-Suess SM. A Study of zoledronic acid as neo-adjuvant, perioperative therapy in patients with resectable pancreatic ductal adenocarcinoma. J Cancer Ther. (2013) 4:797–803. 10.4236/jct.2013.43096 24089656 PMC3786568

[56] Ferretti G Fabi A Carlini P Papaldo P Cordiali Fei P Di Cosimo S. Zoledronic-acid-induced circulating level modifications of angiogenic factors, metalloproteinases and proinflammatory cytokines in metastatic breast cancer patients. Oncology. (2005) 69:35–43. 10.1159/000087286 16088233

[57] Roland CL Lynn KD Toombs JE Dineen SP Udugamasooriya DG Brekken RA. Cytokine levels correlate with immune cell infiltration after anti-VEGF therapy in preclinical mouse models of breast cancer. PLoS ONE. (2009) 4:e7669. 10.1371/journal.pone.0007669 19888452 PMC2766251

[58] Ko JS Zea AH Rini BI Ireland JL Elson P Cohen P. Sunitinib mediates reversal of myeloid-derived suppressor cell accumulation in renal cell carcinoma patients. Clin Cancer Res. (2009) 15:2148–57. 10.1158/1078-0432.CCR-08-1332 19276286

[59] Chen HM Ma G Gildener-Leapman N Eisenstein S Coakley BA Ozao J. Myeloid-derived suppressor cells as an immune parameter in patients with concurrent sunitinib and stereotactic body radiotherapy. Clin Cancer Res. (2015) 21:4073–85. 10.1158/1078-0432.CCR-14-2742 25922428 PMC4720266

[60] Eruslanov E Daurkin I Ortiz J Vieweg J Kusmartsev S. Pivotal advance: tumor-mediated induction of myeloid-derived suppressor cells and M2-polarized macrophages by altering intracellular PGE(2) catabolism in myeloid cells. J Leukoc Biol. (2010) 88:839–48. 10.1189/jlb.1209821 20587738 PMC8454925

[61] Veltman JD Lambers ME van Nimwegen M Hendriks RW Hoogsteden HC Aerts JG. COX-2 inhibition improves immunotherapy and is associated with decreased numbers of myeloid-derived suppressor cells in mesothelioma. Celecoxib influences MDSC function. BMC Cancer. (2010) 10:464. 10.1186/1471-2407-10-464 20804550 PMC2939552

[62] Meyer C Sevko A Ramacher M Bazhin AV Falk CS Osen W. Chronic inflammation promotes myeloid-derived suppressor cell activation blocking antitumor immunity in transgenic mouse melanoma model. Proc Natl Acad Sci USA. (2011) 108:17111–6. 10.1073/pnas.1108121108 21969559 PMC3193202

[63] Serafini P Meckel K Kelso M Noonan K Califano J Koch W. Phosphodiesterase-5 inhibition augments endogenous antitumor immunity by reducing myeloid-derived suppressor cell function. J Exp Med. (2006) 203:2691–702. 10.1084/jem.20061104 17101732 PMC2118163

[64] Lin S Wang J Wang L Wen J Guo Y Qiao W. Phosphodiesterase-5 inhibition suppresses colonic inflammation-induced tumorigenesis via blocking the recruitment of MDSC. Am J Cancer Res. (2017) 7:41–52. 28123846 PMC5250679

[65] Califano JA Khan Z Noonan KA Rudraraju L Zhang Z Wang H. Correction: tadalafil augments tumor-specific immunity in patients with head and neck squamous cell carcinoma. Clin Cancer Res. (2018) 24:6100. 10.1158/1078-0432.CCR-18-3298 30510089

[66] Weed DT Vella JL Reis IM De la Fuente AC Gomez C Sargi Z. Tadalafil reduces myeloid-derived suppressor cells and regulatory T cells and promotes tumor immunity in patients with head and neck squamous cell carcinoma. Clin Cancer Res. (2015) 21:39–48. 10.1158/1078-0432.CCR-14-1711 25320361 PMC4322895

[67] Hassel JC Jiang H Bender C Winkler J Sevko A Shevchenko I. Tadalafil has biologic activity in human melanoma. Results of a pilot trial with Tadalafil in patients with metastatic melanoma (TaMe). Oncoimmunology. (2017) 6:e1326440. 10.1080/2162402X.2017.1326440 28932631 PMC5599085

[68] Ali K Soond DR Pineiro R Hagemann T Pearce W Lim EL. Inactivation of PI(3)K p110delta breaks regulatory T-cell-mediated immune tolerance to cancer. Nature. (2014) 510:407–11. 10.1038/nature13444 24919154 PMC4501086

[69] Nagaraj S Youn JI Weber H Iclozan C Lu L Cotter MJ. Anti-inflammatory triterpenoid blocks immune suppressive function of MDSCs and improves immune response in cancer. Clin Cancer Res. (2010) 16:1812–23. 10.1158/1078-0432.CCR-09-3272 20215551 PMC2840181

[70] Wang YY Yang YX Zhe H He ZX Zhou SF. Bardoxolone methyl (CDDO-Me) as a therapeutic agent: an update on its pharmacokinetic and pharmacodynamic properties. Drug Des Devel Ther. (2014) 8:2075–88. 10.2147/DDDT.S68872 25364233 PMC4211867

[71] Li Y Wongsiriroj N Blaner WS. The multifaceted nature of retinoid transport and metabolism. Hepatobiliary Surg Nutr. (2014) 3:126–39. 10.3978/j.issn.2304-3881.2014.05.04 25019074 PMC4073323

[72] Kim K Skora AD Li Z Liu Q Tam AJ Blosser RL. Eradication of metastatic mouse cancers resistant to immune checkpoint blockade by suppression of myeloid-derived cells. Proc Natl Acad Sci USA. (2014) 111:11774–9. 10.1073/pnas.1410626111 25071169 PMC4136565

[73] Orillion A Hashimoto A Damayanti N Shen L Adelaiye-Ogala R Arisa S. Entinostat neutralizes myeloid-derived suppressor cells and enhances the antitumor effect of PD-1 inhibition in murine models of lung and renal cell carcinoma. Clin Cancer Res. (2017) 23:5187–201. 10.1158/1078-0432.CCR-17-0741 28698201 PMC5723438

[74] Christmas BJ Rafie CI Hopkins AC Scott BA Ma HS Cruz KA. Entinostat converts immune-resistant breast and pancreatic cancers into checkpoint-responsive tumors by reprogramming tumor-infiltrating MDSCs. Cancer Immunol Res. (2018) 6:1561–77. 10.1158/2326-6066.CIR-18-0070 30341213 PMC6279584

[75] Hashimoto A Fukumoto T Zhang R Gabrilovich D. Selective targeting of different populations of myeloid-derived suppressor cells by histone deacetylase inhibitors. Cancer Immunol Immunother. (2020). 10.1007/s00262-020-02588-7. [Epub ahead of print]. 32435850 PMC7765083

[76] Wong ALA Hirpara JL Pervaiz S Eu JQ Sethi G Goh BC. Do STAT3 inhibitors have potential in the future for cancer therapy? Expert Opin Investig Drugs. (2017) 26:883–7. 10.1080/13543784.2017.1351941 28714740

[77] Reilley MJ McCoon P Cook C Lyne P Kurzrock R Kim Y. STAT3 antisense oligonucleotide AZD9150 in a subset of patients with heavily pretreated lymphoma: results of a phase 1b trial. J Immunother Cancer. (2018) 6:119. 10.1186/s40425-018-0436-5 30446007 PMC6240242

[78] Iclozan C Antonia S Chiappori A Chen DT Gabrilovich D. Therapeutic regulation of myeloid-derived suppressor cells and immune response to cancer vaccine in patients with extensive stage small cell lung cancer. Cancer Immunol Immunother. (2013) 62:909–18. 10.1007/s00262-013-1396-8 23589106 PMC3662237

[79] Mirza N Fishman M Fricke I Dunn M Neuger AM Frost TJ. All-trans-retinoic acid improves differentiation of myeloid cells and immune response in cancer patients. Cancer Res. (2006) 66:9299–307. 10.1158/0008-5472.CAN-06-1690 16982775 PMC1586106

[80] Nefedova Y Fishman M Sherman S Wang X Beg AA Gabrilovich DI. Mechanism of all-trans retinoic acid effect on tumor-associated myeloid-derived suppressor cells. Cancer Res. (2007) 67:11021–8. 10.1158/0008-5472.CAN-07-2593 18006848

[81] Lathers DM Clark JI Achille NJ Young MR. Phase 1B study to improve immune responses in head and neck cancer patients using escalating doses of 25-hydroxyvitamin D3. Cancer Immunol Immunother. (2004) 53:422–30. 10.1007/s00262-003-0459-7 14648070 PMC11032934

[82] Gabriele L Ozato K. The role of the interferon regulatory factor (IRF) family in dendritic cell development and function. Cytokine Growth Factor Rev. (2007) 18:503–10. 10.1016/j.cytogfr.2007.06.008 17702640

[83] Netherby CS Abrams SI. Mechanisms overseeing myeloid-derived suppressor cell production in neoplastic disease. Cancer Immunol Immunother. (2017) 66:989–96. 10.1007/s00262-017-1963-5 28224211 PMC5522637

[84] Nefedova Y Nagaraj S Rosenbauer A Muro-Cacho C Sebti SM Gabrilovich DI. Regulation of dendritic cell differentiation and antitumor immune response in cancer by pharmacologic-selective inhibition of the janus-activated kinase 2/signal transducers and activators of transcription 3 pathway. Cancer Res. (2005) 65:9525–35. 10.1158/0008-5472.CAN-05-0529 16230418 PMC1351362

[85] Kodumudi KN Woan K Gilvary DL Sahakian E Wei S Djeu JY. A novel chemoimmunomodulating property of docetaxel: suppression of myeloid-derived suppressor cells in tumor bearers. Clin Cancer Res. (2010) 16:4583–94. 10.1158/1078-0432.CCR-10-0733 20702612 PMC3874864

[86] Bray F Ferlay J Soerjomataram I Siegel RL Torre LA Jemal A. Global cancer statistics 2018: GLOBOCAN estimates of incidence and mortality worldwide for 36 cancers in 185 countries. CA Cancer J Clin. (2018) 68:394–424. 10.3322/caac.21492 30207593

[87] Althuis MD Fergenbaum JH Garcia-Closas M Brinton LA Madigan MP Sherman ME. Etiology of hormone receptor-defined breast cancer: a systematic review of the literature. Cancer Epidemiol Biomarkers Prev. (2004) 13:1558–68. 10.1016/S1047-2797(03)00136-4 15466970

[88] Gradishar WJ Anderson BO Balassanian R Blair SL Burstein HJ Cyr A. Invasive breast cancer version 1.2016, NCCN clinical practice guidelines in oncology. J Natl Compr Canc Netw. (2016) 14:324–54. 10.6004/jnccn.2016.0037 26957618

[89] Miller LD Chou JA Black MA Print C Chifman J Alistar A. Immunogenic subtypes of breast cancer delineated by gene classifiers of immune responsiveness. Cancer Immunol Res. (2016) 4:600–10. 10.1158/2326-6066.CIR-15-0149 27197066 PMC4930674

[90] Bates JP Derakhshandeh R Jones L Webb TJ. Mechanisms of immune evasion in breast cancer. BMC Cancer. (2018) 18:556. 10.1186/s12885-018-4441-3 29751789 PMC5948714

[91] Diaz-Montero CM Salem ML Nishimura MI Garrett-Mayer E Cole DJ Montero AJ. Increased circulating myeloid-derived suppressor cells correlate with clinical cancer stage, metastatic tumor burden, and doxorubicin-cyclophosphamide chemotherapy. Cancer Immunol Immunother. (2009) 58:49–59. 10.1007/s00262-008-0523-4 18446337 PMC3401888

[92] Bergenfelz C Larsson AM von Stedingk K Gruvberger-Saal S Aaltonen K Jansson S. Systemic monocytic-MDSCs are generated from monocytes and correlate with disease progression in breast cancer patients. PLoS ONE. (2015) 10:e0127028. 10.1371/journal.pone.0127028 25992611 PMC4439153

[93] Denkert C Loibl S Noske A Roller M Muller BM Komor M. Tumor-associated lymphocytes as an independent predictor of response to neoadjuvant chemotherapy in breast cancer. J Clin Oncol. (2010) 28:105–13. 10.1200/JCO.2009.23.7370 19917869

[94] Denkert C von Minckwitz G Brase JC Sinn BV Gade S Kronenwett R Tumor-infiltrating lymphocytes and response to neoadjuvant chemotherapy with or without carboplatin in human epidermal growth factor receptor 2-positive and triple-negative primary breast cancers. J Clin Oncol. (2015) 33:983–91. 10.1200/JCO.2014.58.1967 25534375

[95] Loi S Michiels S Salgado R Sirtaine N Jose V Fumagalli D. Tumor infiltrating lymphocytes are prognostic in triple negative breast cancer and predictive for trastuzumab benefit in early breast cancer: results from the FinHER trial. Ann Oncol. (2014) 25:1544–50. 10.1093/annonc/mdu112 24608200

[96] Adams S Gray RJ Demaria S Goldstein L Perez EA Shulman LN. Prognostic value of tumor-infiltrating lymphocytes in triple-negative breast cancers from two phase III randomized adjuvant breast cancer trials: ECOG 2197 and ECOG 1199. J Clin Oncol. (2014) 32:2959–66. 10.1200/JCO.2013.55.0491 25071121 PMC4162494

[97] Boutte AM McDonald WH Shyr Y Yang L Lin PC. Characterization of the MDSC proteome associated with metastatic murine mammary tumors using label-free mass spectrometry and shotgun proteomics. PLoS ONE. (2011) 6:e22446. 10.1371/journal.pone.0022446 21853032 PMC3154190

[98] Danilin S Merkel AR Johnson JR Johnson RW Edwards JR Sterling JA. Myeloid-derived suppressor cells expand during breast cancer progression and promote tumor-induced bone destruction. Oncoimmunology. (2012) 1:1484–94. 10.4161/onci.21990 23264895 PMC3525604

[99] Donkor MK Lahue E Hoke TA Shafer LR Coskun U Solheim JC. Mammary tumor heterogeneity in the expansion of myeloid-derived suppressor cells. Int Immunopharmacol. (2009) 9:937–48. 10.1016/j.intimp.2009.03.021 19362167

[100] Le HK Graham L Cha E Morales JK Manjili MH Bear HD. Gemcitabine directly inhibits myeloid derived suppressor cells in BALB/c mice bearing 4T1 mammary carcinoma and augments expansion of T cells from tumor-bearing mice. Int Immunopharmacol. (2009) 9:900–9. 10.1016/j.intimp.2009.03.015 19336265

[101] Mundy-Bosse BL Lesinski GB Jaime-Ramirez AC Benninger K Khan M Kuppusamy P. Myeloid-derived suppressor cell inhibition of the IFN response in tumor-bearing mice. Cancer Res. (2011) 71:5101–10. 10.1158/0008-5472.CAN-10-2670 21680779 PMC3148319

[102] Solito S Falisi E Diaz-Montero CM Doni A Pinton L Rosato A. A human promyelocytic-like population is responsible for the immune suppression mediated by myeloid-derived suppressor cells. Blood. (2011) 118:2254–65. 10.1182/blood-2010-12-325753 21734236 PMC3709641

[103] Yu J Du W Yan F Wang Y Li H Cao S Myeloid-derived suppressor cells suppress antitumor immune responses through IDO expression and correlate with lymph node metastasis in patients with breast cancer. J Immunol. (2013) 190:3783–97. 10.4049/jimmunol.1201449 23440412

[104] Gonda K Shibata M Ohtake T Matsumoto Y Tachibana K Abe N. Myeloid-derived suppressor cells are increased and correlated with type 2 immune responses, malnutrition, inflammation, and poor prognosis in patients with breast cancer. Oncol Lett. (2017) 14:1766–74. 10.3892/ol.2017.6305 28789407 PMC5529875

[105] Safarzadeh E Hashemzadeh S Duijf PHG Mansoori B Khaze V Mohammadi A. Circulating myeloid-derived suppressor cells: an independent prognostic factor in patients with breast cancer. J Cell Physiol. (2019) 234:3515–25. 10.1002/jcp.26896 30362521

[106] Speigl L Burow H Bailur JK Janssen N Walter CB Pawelec G. CD14+ HLA-DR-/low MDSCs are elevated in the periphery of early-stage breast cancer patients and suppress autologous T cell proliferation. Breast Cancer Res Treat. (2018) 168:401–11. 10.1007/s10549-017-4594-9 29230664

[107] Toor SM Syed Khaja AS El Salhat H Faour I Kanbar J Quadri AA. Myeloid cells in circulation and tumor microenvironment of breast cancer patients. Cancer Immunol Immunother. (2017) 66:753–64. 10.1007/s00262-017-1977-z 28283696 PMC5445142

[108] Shou D Wen L Song Z Yin J Sun Q Gong W. Suppressive role of myeloid-derived suppressor cells (MDSCs) in the microenvironment of breast cancer and targeted immunotherapies. Oncotarget. (2016) 7:64505–11. 10.18632/oncotarget.11352 27542274 PMC5325458

[109] Lu T Ramakrishnan R Altiok S Youn JI Cheng P Celis E. Tumor-infiltrating myeloid cells induce tumor cell resistance to cytotoxic T cells in mice. J Clin Invest. (2011) 121:4015–29. 10.1172/JCI45862 21911941 PMC3195459

[110] Li R Wei F Yu J Li H Ren X Hao X. IDO inhibits T-cell function through suppressing Vav1 expression and activation. Cancer Biol Ther. (2009) 8:1402–8. 10.4161/cbt.8.14.8882 19597340

[111] Sun J Yu J Li H Yang L Wei F Yu W. Upregulated expression of indoleamine 2, 3-dioxygenase in CHO cells induces apoptosis of competent T cells and increases proportion of Treg cells. J Exp Clin Cancer Res. (2011) 30:82. 10.1186/1756-9966-30-82 21917155 PMC3184069

[112] Kumar V Donthireddy L Marvel D Condamine T Wang F Lavilla-Alonso S. Cancer-associated fibroblasts neutralize the anti-tumor effect of CSF1 receptor blockade by inducing PMN-MDSC infiltration of tumors. Cancer Cell. (2017) 32:654–68.e5. 10.1016/j.ccell.2017.10.005 29136508 PMC5827952

[113] Lucas C Barnich N Nguyen HTT. Microbiota, inflammation and colorectal cancer. Int J Mol Sci. (2017) 18:1310. 10.3390/ijms18061310 28632155 PMC5486131

[114] Fakih MG. Metastatic colorectal cancer: current state and future directions. J Clin Oncol. (2015) 33:1809–24. 10.1200/JCO.2014.59.7633 25918280

[115] Jager D Halama N Zornig I Klug P Krauss J Haag GM. Immunotherapy of colorectal cancer. Oncol Res Treat. (2016) 39:346–50. 10.1159/000446713 27259331

[116] Weber R Fleming V Hu X Nagibin V Groth C Altevogt P. Myeloid-derived suppressor cells hinder the anti-cancer activity of immune checkpoint inhibitors. Front Immunol. (2018) 9:1310. 10.3389/fimmu.2018.01310 29942309 PMC6004385

[117] Lakatos PL Lakatos L. Risk for colorectal cancer in ulcerative colitis: changes, causes and management strategies. World J Gastroenterol. (2008) 14:3937–47. 10.3748/wjg.14.3937 18609676 PMC2725331

[118] Katoh H Wang D Daikoku T Sun H Dey SK Dubois RN. CXCR2-expressing myeloid-derived suppressor cells are essential to promote colitis-associated tumorigenesis. Cancer Cell. (2013) 24:631–44. 10.1016/j.ccr.2013.10.009 24229710 PMC3928012

[119] Chun E Lavoie S Michaud M Gallini CA Kim J Soucy G. CCL2 promotes colorectal carcinogenesis by enhancing polymorphonuclear myeloid-derived suppressor cell population and function. Cell Rep. (2015) 12:244–57. 10.1016/j.celrep.2015.06.024 26146082 PMC4620029

[120] Garrett WS. Cancer and the microbiota. Science. (2015) 348:80–6. 10.1126/science.aaa4972 25838377 PMC5535753

[121] Kostic AD Chun E Robertson L Glickman JN Gallini CA Michaud M. *Fusobacterium nucleatum* potentiates intestinal tumorigenesis and modulates the tumor-immune microenvironment. Cell Host Microbe. (2013) 14:207–15. 10.1016/j.chom.2013.07.007 23954159 PMC3772512

[122] De Cicco P Sanders T Cirino G Maloy KJ Ianaro A Hydrogen sulfide reduces myeloid-derived suppressor cell-mediated inflammatory response in a model of *Helicobacter hepaticus*-induced colitis. Front Immunol. (2018) 9:499 10.3389/fimmu.2018.00499 29636751 PMC5880908

[123] Zhang Y Wang J Wang W Tian J Yin K Tang X IL-17A produced by peritoneal macrophages promote the accumulation and function of granulocytic myeloid-derived suppressor cells in the development of colitis-associated cancer. Tumour Biol. (2016) 37:15883–91. 10.1007/s13277-016-5414-2 27909978

[124] Sun HL Zhou X Xue YF Wang K Shen YF Mao JJ. Increased frequency and clinical significance of myeloid-derived suppressor cells in human colorectal carcinoma. World J Gastroenterol. (2012) 18:3303–9. 10.3748/wjg.v18.i25.3303 22783056 PMC3391769

[125] Zhang B Wang Z Wu L Zhang M Li W Ding J. Circulating and tumor-infiltrating myeloid-derived suppressor cells in patients with colorectal carcinoma. PLoS ONE. (2013) 8:e57114. 10.1371/journal.pone.0057114 23437326 PMC3577767

[126] OuYang LY Wu XJ Ye SB Zhang RX Li ZL Liao W. Tumor-induced myeloid-derived suppressor cells promote tumor progression through oxidative metabolism in human colorectal cancer. J Transl Med. (2015) 13:47. 10.1186/s12967-015-0410-7 25638150 PMC4357065

[127] Toor SM Syed Khaja AS El Salhat H Bekdache O Kanbar J Jaloudi M. Increased levels of circulating and tumor-infiltrating granulocytic myeloid cells in colorectal cancer patients. Front Immunol. (2016) 7:560. 10.3389/fimmu.2016.00560 28008330 PMC5143474

[128] Wu P Wu D Ni C Ye J Chen W Hu G. γ*δ*T17 cells promote the accumulation and expansion of myeloid-derived suppressor cells in human colorectal cancer. Immunity. (2014) 40:785–800. 10.1016/j.immuni.2014.03.013 24816404 PMC4716654

[129] Yan J Huang J. Innate γ*δ*T17 cells convert cancer-elicited inflammation into immunosuppression through myeloid-derived suppressor cells. Oncoimmunology. (2014) 3:e953423. 10.4161/21624011.2014.953423 25610744 PMC4292213

[130] Yang R Cai TT Wu XJ Liu YN He J Zhang XS. Tumour YAP1 and PTEN expression correlates with tumour-associated myeloid suppressor cell expansion and reduced survival in colorectal cancer. Immunology. (2018) 155:263–72. 10.1111/imm.12949 29770434 PMC6142285

[131] Garton AJ Seibel S Lopresti-Morrow L Crew L Janson N Mandiyan S. Anti-KIT monoclonal antibody treatment enhances the antitumor activity of immune checkpoint inhibitors by reversing tumor-induced immunosuppression. Mol Cancer Ther. (2017) 16:671–80. 10.1158/1535-7163.MCT-16-0676 28138031

[132] Ye TH Yang FF Zhu YX Li YL Lei Q Song XJ. Inhibition of Stat3 signaling pathway by nifuroxazide improves antitumor immunity and impairs colorectal carcinoma metastasis. Cell Death Dis. (2017) 8:e2534. 10.1038/cddis.2016.452 28055016 PMC5386364

[133] Dominguez GA Condamine T Mony S Hashimoto A Wang F Liu Q. Selective targeting of myeloid-derived suppressor cells in cancer patients using DS-8273a, an agonistic TRAIL-R2 antibody. Clin Cancer Res. (2017) 23:2942–50. 10.1158/1078-0432.CCR-16-1784 27965309 PMC5468499

[134] Ross MI Gershenwald JE. Evidence-based treatment of early-stage melanoma. J Surg Oncol. (2011) 104:341–53. 10.1002/jso.21962 21858828

[135] Lideikaite A Mozuraitiene J Letautiene S. Analysis of prognostic factors for melanoma patients. Acta Med Litu. (2017) 24:25–34. 10.6001/actamedica.v24i1.3460 28630590 PMC5467960

[136] Sharma P Hu-Lieskovan S Wargo JA Ribas A. Primary, adaptive, and acquired resistance to cancer immunotherapy. Cell. (2017) 168:707–23. 10.1016/j.cell.2017.01.017 28187290 PMC5391692

[137] Fujimura T Kambayashi Y Aiba S. Crosstalk between regulatory T cells (Tregs) and myeloid derived suppressor cells (MDSCs) during melanoma growth. Oncoimmunology. (2012) 1:1433–4. 10.4161/onci.21176 23243619 PMC3518528

[138] Filipazzi P Huber V Rivoltini L. Phenotype, function and clinical implications of myeloid-derived suppressor cells in cancer patients. Cancer Immunol Immunother. (2012) 61:255–63. 10.1007/s00262-011-1161-9 22120756 PMC11029611

[139] Poschke I Kiessling R. On the armament and appearances of human myeloid-derived suppressor cells. Clin Immunol. (2012) 144:250–68. 10.1016/j.clim.2012.06.003 22858650

[140] Weide B Martens A Zelba H Stutz C Derhovanessian E Di Giacomo AM Myeloid-derived suppressor cells predict survival of patients with advanced melanoma: comparison with regulatory T cells and NY-ESO-1- or melan-A-specific T cells. Clin Cancer Res. (2014) 20:1601–9. 10.1158/1078-0432.CCR-13-2508 24323899

[141] Filipazzi P Valenti R Huber V Pilla L Canese P Iero M. Identification of a new subset of myeloid suppressor cells in peripheral blood of melanoma patients with modulation by a granulocyte-macrophage colony-stimulation factor-based antitumor vaccine. J Clin Oncol. (2007) 25:2546–53. 10.1200/JCO.2006.08.5829 17577033

[142] Jordan KR Amaria RN Ramirez O Callihan EB Gao D Borakove M. Myeloid-derived suppressor cells are associated with disease progression and decreased overall survival in advanced-stage melanoma patients. Cancer Immunol Immunother. (2013) 62:1711–22. 10.1007/s00262-013-1475-x 24072401 PMC4176615

[143] Stanojevic I Miller K Kandolf-Sekulovic L Mijuskovic Z Zolotarevski L Jovic M. A subpopulation that may correspond to granulocytic myeloid-derived suppressor cells reflects the clinical stage and progression of cutaneous melanoma. Int Immunol. (2016) 28:87–97. 10.1093/intimm/dxv053 26391013 PMC4885218

[144] Fleming V Hu X Weber R Nagibin V Groth C Altevogt P. Targeting myeloid-derived suppressor cells to bypass tumor-induced immunosuppression. Front Immunol. (2018) 9:398. 10.3389/fimmu.2018.00398 29552012 PMC5840207

[145] Anani W Shurin MR. Targeting myeloid-derived suppressor cells in cancer. Adv Exp Med Biol. (2017) 1036:105–28. 10.1007/978-3-319-67577-0_8 29275468

[146] Tobin RP Davis D Jordan KR McCarter MD. The clinical evidence for targeting human myeloid-derived suppressor cells in cancer patients. J Leukoc Biol. (2017) 102:381–91. 10.1189/jlb.5VMR1016-449R 28179538 PMC6608076

[147] Varney ML Johansson SL Singh RK. Distinct expression of CXCL8 and its receptors CXCR1 and CXCR2 and their association with vessel density and aggressiveness in malignant melanoma. Am J Clin Pathol. (2006) 125:209–16. 10.1309/VPL5R3JR7F1D6V03 16393674

[148] Singh S Nannuru KC Sadanandam A Varney ML Singh RK. CXCR1 and CXCR2 enhances human melanoma tumourigenesis, growth and invasion. Br J Cancer. (2009) 100:1638–46. 10.1038/sj.bjc.6605055 19401689 PMC2696769

[149] Sharma B Singh S Varney ML Singh RK. Targeting CXCR1/CXCR2 receptor antagonism in malignant melanoma. Expert Opin Ther Targets. (2010) 14:435–42. 10.1517/14728221003652471 20230195 PMC4229031

[150] Probst BL Trevino I McCauley L Bumeister R Dulubova I Wigley WC. RTA 408, a novel synthetic triterpenoid with broad anticancer and anti-inflammatory activity. PLoS ONE. (2015) 10:e0122942. 10.1371/journal.pone.0122942 25897966 PMC4405374

[151] Creelan BC Gabrilovich DI Gray JE Williams CC Tanvetyanon T Haura EB. Safety, pharmacokinetics, and pharmacodynamics of oral omaveloxolone (RTA 408), a synthetic triterpenoid, in a first-in-human trial of patients with advanced solid tumors. Onco Targets Ther. (2017) 10:4239–50. 10.2147/OTT.S136992 28919776 PMC5587199

[152] Sade-Feldman M Kanterman J Ish-Shalom E Elnekave M Horwitz E Baniyash M. Tumor necrosis factor-alpha blocks differentiation and enhances suppressive activity of immature myeloid cells during chronic inflammation. Immunity. (2013) 38:541–54. 10.1016/j.immuni.2013.02.007 23477736

[153] Sevko A Umansky V. Myeloid-derived suppressor cells interact with tumors in terms of myelopoiesis, tumorigenesis and immunosuppression: thick as thieves. J Cancer. (2013) 4:3–11. 10.7150/jca.5047 23386900 PMC3564242

[154] Botti G Fratangelo F Cerrone M Liguori G Cantile M Anniciello AM. COX-2 expression positively correlates with PD-L1 expression in human melanoma cells. J Transl Med. (2017) 15:46. 10.1186/s12967-017-1150-7 28231855 PMC5324267

[155] Panza E De Cicco P Ercolano G Armogida C Scognamiglio G Anniciello AM. Differential expression of cyclooxygenase-2 in metastatic melanoma affects progression free survival. Oncotarget. (2016) 7:57077–85. 10.18632/oncotarget.10976 27494851 PMC5302974

[156] Goulet AC Einsphar JG Alberts DS Beas A Burk C Bhattacharyya A. Analysis of cyclooxygenase 2 (COX-2) expression during malignant melanoma progression. Cancer Biol Ther. (2003) 2:713–8. 10.4161/cbt.2.6.627 14688483

[157] Ohl K Tenbrock K. Reactive oxygen species as regulators of MDSC-mediated immune suppression. Front Immunol. (2018) 9:2499. 10.3389/fimmu.2018.02499 30425715 PMC6218613

[158] Stiff A Trikha P Mundy-Bosse B McMichael E Mace TA Benner B. nitric oxide production by myeloid-derived suppressor cells plays a role in impairing Fc receptor-mediated natural killer cell function. Clin Cancer Res. (2018) 24:1891–904. 10.1158/1078-0432.CCR-17-0691 29363526 PMC7184799

[159] Vasquez-Dunddel D Pan F Zeng Q Gorbounov M Albesiano E Fu J. STAT3 regulates arginase-I in myeloid-derived suppressor cells from cancer patients. J Clin Invest. (2013) 123:1580–9. 10.1172/JCI60083 23454751 PMC3613901

[160] Kanterman J Sade-Feldman M Baniyash M. New insights into chronic inflammation-induced immunosuppression. Semin Cancer Biol. (2012) 22:307–18. 10.1016/j.semcancer.2012.02.008 22387003

[161] Zelenay S van der Veen AG Bottcher JP Snelgrove KJ Rogers N Acton SE. Cyclooxygenase-dependent tumor growth through evasion of immunity. Cell. (2015) 162:1257–70. 10.1016/j.cell.2015.08.015 26343581 PMC4597191

[162] Ostrand-Rosenberg S. Myeloid-derived suppressor cells: more mechanisms for inhibiting antitumor immunity. Cancer Immunol Immunother. (2010) 59:1593–600. 10.1007/s00262-010-0855-8 20414655 PMC3706261

[163] Ugel S Delpozzo F Desantis G Papalini F Simonato F Sonda N. Therapeutic targeting of myeloid-derived suppressor cells. Curr Opin Pharmacol. (2009) 9:470–81. 10.1016/j.coph.2009.06.014 19616475

[164] De Cicco P Ercolano G Rubino V Terrazzano G Ruggiero G Cirino G. Modulation of the functions of myeloid-derived suppressor cells: a new strategy of hydrogen sulfide anti-cancer effects. Br J Pharmacol. (2020) 177:884–97. 10.1111/bph.14824 31392723 PMC7024705

[165] Chen S Zhang Y Kuzel TM Zhang B. Regulating tumor myeloid-derived suppressor cells by MicroRNAs. Cancer Cell Microenviron. (2015) 2:e637. 10.14800/ccm.637 26005707 PMC4440580

[166] Calin GA Croce CM MicroRNA signatures in human cancers. Nat Rev Cancer. (2006) 6:857–66. 10.1038/nrc1997 17060945

[167] Fattore L Costantini S Malpicci D Ruggiero CF Ascierto PA Croce CM. MicroRNAs in melanoma development and resistance to target therapy. Oncotarget. (2017) 8:22262–78. 10.18632/oncotarget.14763 28118616 PMC5400662

[168] Jansson MD Lund AH. MicroRNA and cancer. Mol Oncol. (2012) 6:590–610. 10.1016/j.molonc.2012.09.006 23102669 PMC5528350

[169] Qian H Yang C Yang Y. MicroRNA-26a inhibits the growth and invasiveness of malignant melanoma and directly targets on MITF gene. Cell Death Discov. (2017) 3:17028. 10.1038/cddiscovery.2017.28 28698805 PMC5502303

[170] Alderman C Sehlaoui A Xiao Z Yang Y. MicroRNA-15a inhibits the growth and invasiveness of malignant melanoma and directly targets on CDCA4 gene. Tumour Biol. (2016) 37:13941–50. 10.1007/s13277-016-5271-z 27492455

[171] Panza E Ercolano G De Cicco P Armogida C Scognamiglio G Botti G. MicroRNA-143–3p inhibits growth and invasiveness of melanoma cells by targeting cyclooxygenase-2 and inversely correlates with malignant melanoma progression. Biochem Pharmacol. (2018) 156:52–9. 10.1016/j.bcp.2018.08.008 30098313

[172] Chen S Wang L Fan J Ye C Dominguez D Zhang Y. Host miR155 promotes tumor growth through a myeloid-derived suppressor cell-dependent mechanism. Cancer Res. (2015) 75:519–31. 10.1158/0008-5472.CAN-14-2331 25502838 PMC4315710

[173] Huber V Vallacchi V Fleming V Hu X Cova A Dugo M. Tumor-derived microRNAs induce myeloid suppressor cells and predict immunotherapy resistance in melanoma. J Clin Invest. (2018) 128:5505–16. 10.1172/JCI98060 30260323 PMC6264733

[174] Siegel RL Miller KD Jemal A Cancer statistics, 2017. CA Cancer J Clin. (2017) 67:7–30. 10.3322/caac.21387 28055103

[175] Epstein JI Egevad L Amin MB Delahunt B Srigley JR Humphrey PA. The 2014 international society of urological pathology (ISUP) consensus conference on gleason grading of prostatic carcinoma: definition of grading patterns and proposal for a new grading system. Am J Surg Pathol. (2016) 40:244–52. 10.1097/PAS.0000000000000530 26492179

[176] Sharifi N Gulley JL Dahut WL. Androgen deprivation therapy for prostate cancer. JAMA. (2005) 294:238–44. 10.1001/jama.294.2.238 16014598

[177] Yap TA Smith AD Ferraldeschi R Al-Lazikani B Workman P de Bono JS. Drug discovery in advanced prostate cancer: translating biology into therapy. Nat Rev Drug Discov. (2016) 15:699–718. 10.1038/nrd.2016.120 27444228

[178] Wadosky KM Koochekpour S. Molecular mechanisms underlying resistance to androgen deprivation therapy in prostate cancer. Oncotarget. (2016) 7:64447–70. 10.18632/oncotarget.10901 27487144 PMC5325456

[179] Sumanasuriya S De Bono J. Treatment of advanced prostate cancer-a review of current therapies and future promise. Cold Spring Harb Perspect Med. (2018) 8:a030635. 10.1101/cshperspect.a030635 29101113 PMC5983161

[180] Tran C Ouk S Clegg NJ Chen Y Watson PA Arora V. Development of a second-generation antiandrogen for treatment of advanced prostate cancer. Science. (2009) 324:787–90. 10.1126/science.1168175 19359544 PMC2981508

[181] Attard G Belldegrun AS de Bono JS. Selective blockade of androgenic steroid synthesis by novel lyase inhibitors as a therapeutic strategy for treating metastatic prostate cancer. BJU Int. (2005) 96:1241–6. 10.1111/j.1464-410X.2005.05821.x 16287438

[182] Anassi E Ndefo UA. Sipuleucel-T (provenge) injection: the first immunotherapy agent (vaccine) for hormone-refractory prostate cancer. P T. (2011) 36:197–202. 21572775 PMC3086121

[183] Chandrasekar T Yang JC Gao AC Evans CP. Mechanisms of resistance in castration-resistant prostate cancer (CRPC). Transl Androl Urol. (2015) 4:365–80. 10.3978/j.issn.2223-4683.2015.05.02 26814148 PMC4708226

[184] Shi J Wang L Zou C Xia Y Qin S Keller E. Tumor microenvironment promotes prostate cancer cell dissemination via the Akt/mTOR pathway. Oncotarget. (2018) 9:9206–18. 10.18632/oncotarget.24104 29507684 PMC5823632

[185] Corn PG. The tumor microenvironment in prostate cancer: elucidating molecular pathways for therapy development. Cancer Manag Res. (2012) 4:183–93. 10.2147/CMAR.S32839 22904640 PMC3421469

[186] Calcinotto A Spataro C Zagato E Di Mitri D Gil V Crespo M. IL-23 secreted by myeloid cells drives castration-resistant prostate cancer. Nature. (2018) 559:363–9. 10.1038/s41586-018-0266-0 29950727 PMC6461206

[187] Alimonti A Calcinotto A Morilla A Sharp A Bianchini D Boysen G Myeloid-derived suppressor cells (MDSCs) in metastatic castration-resistant prostate cancer (CRPC) patients (PTS). Ann Oncol. (2016) 27:243–65. 10.1093/annonc/mdw372.41

[188] Di Mitri D Toso A Chen JJ Sarti M Pinton S Jost TR. Tumour-infiltrating Gr-1+ myeloid cells antagonize senescence in cancer. Nature. (2014) 515:134–7. 10.1038/nature13638 25156255

[189] Nuhn P Vaghasia AM Goyal J Zhou XC Carducci MA Eisenberger MA. Association of pretreatment neutrophil-to-lymphocyte ratio (NLR) and overall survival (OS) in patients with metastatic castration-resistant prostate cancer (mCRPC) treated with first-line docetaxel. BJU Int. (2014) 114:E11–7. 10.1111/bju.12531 24529213 PMC4004702

[190] Chi N Tan Z Ma K Bao L Yun Z. Increased circulating myeloid-derived suppressor cells correlate with cancer stages, interleukin-8 and−6 in prostate cancer. Int J Clin Exp Med. (2014) 7:3181–92. 25419348 PMC4238489

[191] Idorn M Kollgaard T Kongsted P Sengelov L Thor Straten P. Correlation between frequencies of blood monocytic myeloid-derived suppressor cells, regulatory T cells and negative prognostic markers in patients with castration-resistant metastatic prostate cancer. Cancer Immunol Immunother. (2014) 63:1177–87. 10.1007/s00262-014-1591-2 25085000 PMC11028426

[192] Vasto S Carruba G Candore G Italiano E Di Bona D Caruso C. Inflammation and prostate cancer. Future Oncol. (2008) 4:637–45. 10.2217/14796694.4.5.637 18922121

[193] Chung TD Yu JJ Spiotto MT Bartkowski M Simons JW. Characterization of the role of IL-6 in the progression of prostate cancer. Prostate. (1999) 38:199–207. 10.1002/(SICI)1097-0045(19990215)38:3<199::AID-PROS4>3.0.CO;2-H 10068344

[194] Alcover J Filella X Luque P Molina R Izquierdo L Auge JM. Prognostic value of IL-6 in localized prostatic cancer. Anticancer Res. (2010) 30:4369–72. 21036766

[195] Wu CT Hsieh CC Lin CC Chen WC Hong JH Chen MF. Significance of IL-6 in the transition of hormone-resistant prostate cancer and the induction of myeloid-derived suppressor cells. J Mol Med. (2012) 90:1343–55. 10.1007/s00109-012-0916-x 22660275

[196] Ara T Declerck YA. Interleukin-6 in bone metastasis and cancer progression. Eur J Cancer. (2010) 46:1223–31. 10.1016/j.ejca.2010.02.026 20335016 PMC2917917

[197] Lopez-Bujanda Z Drake CG. Myeloid-derived cells in prostate cancer progression: phenotype and prospective therapies. J Leukoc Biol. (2017) 102:393–406. 10.1189/jlb.5VMR1116-491RR 28550116 PMC6608078

[198] Chen H Libertini SJ George M Dandekar S Tepper CG Al-Bataina B. Genome-wide analysis of androgen receptor binding and gene regulation in two CWR22-derived prostate cancer cell lines. Endocr Relat Cancer. (2010) 17:857–73. 10.1677/ERC-10-0081 20634343 PMC3539310

[199] Isaacs JT. The long and winding road for the development of tasquinimod as an oral second-generation quinoline-3-carboxamide antiangiogenic drug for the treatment of prostate cancer. Expert Opin Investig Drugs. (2010) 19:1235–43. 10.1517/13543784.2010.514262 20836618 PMC4124623

[200] Sunahori K Yamamura M Yamana J Takasugi K Kawashima M Yamamoto H. The S100A8/A9 heterodimer amplifies proinflammatory cytokine production by macrophages via activation of nuclear factor kappa B and p38 mitogen-activated protein kinase in rheumatoid arthritis. Arthritis Res Ther. (2006) 8:R69. 10.1186/ar1939 16613612 PMC1526633

[201] Sinha P Okoro C Foell D Freeze HH Ostrand-Rosenberg S Srikrishna G. Proinflammatory S100 proteins regulate the accumulation of myeloid-derived suppressor cells. J Immunol. (2008) 181:4666–75. 10.4049/jimmunol.181.7.4666 18802069 PMC2810501

[202] Armstrong AJ Haggman M Stadler WM Gingrich JR Assikis V Polikoff J. Long-term survival and biomarker correlates of tasquinimod efficacy in a multicenter randomized study of men with minimally symptomatic metastatic castration-resistant prostate cancer. Clin Cancer Res. (2013) 19:6891–901. 10.1158/1078-0432.CCR-13-1581 24255071 PMC4251453

[203] Armstrong AJ Kaboteh R Carducci MA Damber JE Stadler WM Hansen M. Assessment of the bone scan index in a randomized placebo-controlled trial of tasquinimod in men with metastatic castration-resistant prostate cancer (mCRPC). Urol Oncol. (2014) 32:1308–16. 10.1016/j.urolonc.2014.08.006 25240761 PMC6341998

[204] Sternberg C Armstrong A Pili R Ng S Huddart R Agarwal N. Randomized, Double-blind, placebo-controlled phase III study of tasquinimod in men with metastatic castration-resistant prostate cancer. J Clin Oncol. (2016) 34:2636–43. 10.1200/JCO.2016.66.9697 27298414

[205] Rawla P Sunkara T Muralidharan P Raj JP. Update in global trends and aetiology of hepatocellular carcinoma. Contemp Oncol. (2018) 22:141–50. 10.5114/wo.2018.78941 30455585 PMC6238087

[206] Llovet JM Ricci S Mazzaferro V Hilgard P Gane E Blanc JF. Sorafenib in advanced hepatocellular carcinoma. N Engl J Med. (2008) 359:378–90. 10.1056/NEJMoa0708857 18650514

[207] Marrero JA Kulik LM Sirlin CB Zhu AX Finn RS Abecassis MM. Diagnosis, staging, and management of hepatocellular carcinoma: 2018 practice guidance by the American association for the study of liver diseases. Hepatology. (2018) 68:723–50. 10.1002/hep.29913 29624699

[208] Bruix J Qin S Merle P Granito A Huang YH Bodoky G. Regorafenib for patients with hepatocellular carcinoma who progressed on sorafenib treatment (RESORCE): a randomised, double-blind, placebo-controlled, phase 3 trial. Lancet. (2017) 389:56–66. 10.1016/S0140-6736(16)32453-9 27932229

[209] Matzner Y Bar-Ner M Yahalom J Ishai-Michaeli R Fuks Z Vlodavsky I. Degradation of heparan sulfate in the subendothelial extracellular matrix by a readily released heparanase from human neutrophils. Possible role in invasion through basement membranes. J Clin Invest. (1985) 76:1306–13. 10.1172/JCI112104 2997275 PMC424062

[210] Johnston MP Khakoo SI. Immunotherapy for hepatocellular carcinoma: current and future. World J Gastroenterol. (2019) 25:2977–89. 10.3748/wjg.v25.i24.2977 31293335 PMC6603808

[211] Zhu AX Finn RS Edeline J Cattan S Ogasawara S Palmer D. Pembrolizumab in patients with advanced hepatocellular carcinoma previously treated with sorafenib (KEYNOTE-224): a non-randomised, open-label phase 2 trial. Lancet Oncol. (2018) 19:940–52. 10.1016/S1470-2045(18)30351-6 29875066

[212] Sia D Jiao Y Martinez-Quetglas I Kuchuk O Villacorta-Martin C Castro de Moura M. Identification of an immune-specific class of hepatocellular carcinoma, based on molecular features. Gastroenterology. (2017) 153:812–26. 10.1053/j.gastro.2017.06.007 28624577 PMC12166766

[213] Hoechst B Ormandy LA Ballmaier M Lehner F Kruger C Manns MP. A new population of myeloid-derived suppressor cells in hepatocellular carcinoma patients induces CD4^+^CD25^+^Foxp3^+^ T cells. Gastroenterology. (2008) 135:234–43. 10.1053/j.gastro.2008.03.020 18485901

[214] Roth GS Decaens T. Liver immunotolerance and hepatocellular carcinoma: Patho-physiological mechanisms and therapeutic perspectives. Eur J Cancer. (2017) 87:101–12. 10.1016/j.ejca.2017.10.010 29145036

[215] Ringelhan M Pfister D O'Connor T Pikarsky E Heikenwalder M. The immunology of hepatocellular carcinoma. Nat Immunol. (2018) 19:222–32. 10.1038/s41590-018-0044-z 29379119

[216] Li X Xing YF Lei AH Xiao Q Lin ZH Hong YF. Neutrophil count is associated with myeloid derived suppressor cell level and presents prognostic value of for hepatocellular carcinoma patients. Oncotarget. (2017) 8:24380–8. 10.18632/oncotarget.15456 28412745 PMC5421855

[217] Zhou J Liu M Sun H Feng Y Xu L Chan AWH. Hepatoma-intrinsic CCRK inhibition diminishes myeloid-derived suppressor cell immunosuppression and enhances immune-checkpoint blockade efficacy. Gut. (2018) 67:931–44. 10.1136/gutjnl-2017-314032 28939663 PMC5961939

[218] Elwan N Salem ML Kobtan A El-Kalla F Mansour L Yousef M. High numbers of myeloid derived suppressor cells in peripheral blood and ascitic fluid of cirrhotic and HCC patients. Immunol Invest. (2018) 47:169–80. 10.1080/08820139.2017.1407787 29182438

[219] Gao XH Tian L Wu J Ma XL Zhang CY Zhou Y. Circulating CD14^+^ HLA-DR(-/low) myeloid-derived suppressor cells predicted early recurrence of hepatocellular carcinoma after surgery. Hepatol Res. (2017) 47:1061–71. 10.1111/hepr.12831 27764536

[220] Arihara F Mizukoshi E Kitahara M Takata Y Arai K Yamashita T. Increase in CD14+HLA-DR -/low myeloid-derived suppressor cells in hepatocellular carcinoma patients and its impact on prognosis. Cancer Immunol Immunother. (2013) 62:1421–30. 10.1007/s00262-013-1447-1 23764929 PMC11029267

[221] Hetta HF Zahran AM Mansor SG Abdel-Malek MO Mekky MA Abbas WA. Frequency and Implications of myeloid-derived suppressor cells and lymphocyte subsets in Egyptian patients with hepatitis C virus-related hepatocellular carcinoma. J Med Virol. (2019) 91:1319–28. 10.1002/jmv.25428 30761547

[222] Nan J Xing YF Hu B Tang JX Dong HM He YM. Endoplasmic reticulum stress induced LOX-1^+^ CD15^+^ polymorphonuclear myeloid-derived suppressor cells in hepatocellular carcinoma. Immunology. (2018) 154:144–55. 10.1111/imm.12876 29211299 PMC5904716

[223] Hoechst B Voigtlaender T Ormandy L Gamrekelashvili J Zhao F Wedemeyer H. Myeloid derived suppressor cells inhibit natural killer cells in patients with hepatocellular carcinoma via the NKp30 receptor. Hepatology. (2009) 50:799–807. 10.1002/hep.23054 19551844 PMC6357774

[224] Shen P Wang A He M Wang Q Zheng S. Increased circulating Lin(-/low) CD33^+^ HLA-DR^−^ myeloid-derived suppressor cells in hepatocellular carcinoma patients. Hepatol Res. (2014) 44:639–50. 10.1111/hepr.12167 23701406

[225] Yu H Kortylewski M Pardoll D. Crosstalk between cancer and immune cells: role of STAT3 in the tumour microenvironment. Nat Rev Immunol. (2007) 7:41–51. 10.1038/nri1995 17186030

[226] Kapanadze T Gamrekelashvili J Ma C Chan C Zhao F Hewitt S. Regulation of accumulation and function of myeloid derived suppressor cells in different murine models of hepatocellular carcinoma. J Hepatol. (2013) 59:1007–13. 10.1016/j.jhep.2013.06.010 23796475 PMC3805787

[227] Cao M Xu Y Youn JI Cabrera R Zhang X Gabrilovich D. Kinase inhibitor Sorafenib modulates immunosuppressive cell populations in a murine liver cancer model. Lab Invest. (2011) 91:598–608. 10.1038/labinvest.2010.205 21321535 PMC3711234

[228] Chang CJ Yang YH Chiu CJ Lu LC Liao CC Liang CW. Targeting tumor-infiltrating Ly6G^+^ myeloid cells improves sorafenib efficacy in mouse orthotopic hepatocellular carcinoma. Int J Cancer. (2018) 142:1878–89. 10.1002/ijc.31216 29266245

[229] Chen Y Huang Y Reiberger T Duyverman AM Huang P Samuel R. Differential effects of sorafenib on liver versus tumor fibrosis mediated by stromal-derived factor 1 alpha/C-X-C receptor type 4 axis and myeloid differentiation antigen-positive myeloid cell infiltration in mice. Hepatology. (2014) 59:1435–47. 10.1002/hep.26790 24242874 PMC3966948

[230] Chiu DK Tse AP Xu IM Di Cui J Lai RK Li LL. Hypoxia inducible factor HIF-1 promotes myeloid-derived suppressor cells accumulation through ENTPD2/CD39L1 in hepatocellular carcinoma. Nat Commun. (2017) 8:517. 10.1038/s41467-017-00530-7 28894087 PMC5593860

[231] Motoshima T Komohara Y Horlad H Takeuchi A Maeda Y Tanoue K. Sorafenib enhances the antitumor effects of anti-CTLA-4 antibody in a murine cancer model by inhibiting myeloid-derived suppressor cells. Oncol Rep. (2015) 33:2947–53. 10.3892/or.2015.3893 25845968

[232] Chen J Wang Z Ding Y Huang F Huang W Lan R. Hypofractionated irradiation suppressed the off-target mouse hepatocarcinoma growth by inhibiting myeloid-derived suppressor cell-mediated immune suppression. Front Oncol. (2020) 10:4. 10.3389/fonc.2020.00004 32117702 PMC7026455

[233] Siegel RL Miller KD Jemal A Cancer statistics, 2019. CA Cancer J Clin. (2019) 69:7–34. 10.3322/caac.21551 30620402

[234] Karim SM Zekri J. Chemotherapy for small cell lung cancer: a comprehensive review. Oncol Rev. (2012) 6:e4. 10.4081/oncol.2012.e4 25992206 PMC4419639

[235] Li D He S. Pemetrexed and cyclophosphamide combination therapy for the treatment of non-small cell lung cancer. Int J Clin Exp Pathol. (2015) 8:14693–700. 26823793 PMC4713579

[236] Schiller JH. Current standards of care in small-cell and non-small-cell lung cancer. Oncology. (2001) 61(Suppl. 1):3–13. 10.1159/000055386 11598409

[237] Silva AP Coelho PV Anazetti M Simioni PU. Targeted therapies for the treatment of non-small-cell lung cancer: monoclonal antibodies and biological inhibitors. Hum Vaccin Immunother. (2017) 13:843–53. 10.1080/21645515.2016.1249551 27831000 PMC5404364

[238] Zhou L Wang XL Deng QL Du YQ Zhao NQ. The efficacy and safety of immunotherapy in patients with advanced NSCLC: a systematic review and meta-analysis. Sci Rep. (2016) 6:32020. 10.1038/srep32020 27558285 PMC4997317

[239] Hirsch FR Scagliotti GV Mulshine JL Kwon R Curran WJ Jr Wu Y-J. Lung cancer: current therapies and new targeted treatments. Lancet. (2017) 389:299–311. 10.1016/S0140-6736(16)30958-8 27574741

[240] Regzedmaa O Zhang H Liu H Chen J. Immune checkpoint inhibitors for small cell lung cancer: opportunities and challenges. Onco Targets Ther. (2019) 12:4605–20. 10.2147/OTT.S204577 31354294 PMC6580132

[241] Milette S Fiset PO Walsh LA Spicer JD Quail DF. The innate immune architecture of lung tumors and its implication in disease progression. J Pathol. (2019) 247:589–605. 10.1002/path.5241 30680732

[242] Huang A Zhang B Wang B Zhang F Fan KX Guo YJ. Increased CD14^+^HLA-DR (-/low) myeloid-derived suppressor cells correlate with extrathoracic metastasis and poor response to chemotherapy in non-small cell lung cancer patients. Cancer Immunol Immunother. (2013) 62:1439–51. 10.1007/s00262-013-1450-6 23760662 PMC11028777

[243] Liu CY Wang YM Wang CL Feng PH Ko HW Liu YH. Population alterations of L-arginase- and inducible nitric oxide synthase-expressed CD11b+/CD14(-)/CD15+/CD33+ myeloid-derived suppressor cells and CD8+ T lymphocytes in patients with advanced-stage non-small cell lung cancer. J Cancer Res Clin Oncol. (2010) 136:35–45. 10.1007/s00432-009-0634-0 19572148 PMC11827779

[244] Feng PH Lee KY Chang YL Chan YF Kuo LW Lin TY. CD14^+^S100A9^+^ monocytic myeloid-derived suppressor cells and their clinical relevance in non-small cell lung cancer. Am J Respir Crit Care Med. (2012) 186:1025–36. 10.1164/rccm.201204-0636OC 22955317 PMC4132576

[245] Heuvers ME Muskens F Bezemer K Lambers M Dingemans AC Groen HJM. Arginase-1 mRNA expression correlates with myeloid-derived suppressor cell levels in peripheral blood of NSCLC patients. Lung Cancer. (2013) 81:468–74. 10.1016/j.lungcan.2013.06.005 23850196

[246] de Goeje PL Bezemer K Heuvers ME Dingemans AC Groen HJ Smit EF. Immunoglobulin-like transcript 3 is expressed by myeloid-derived suppressor cells and correlates with survival in patients with non-small cell lung cancer. Oncoimmunology. (2015) 4:e1014242. 10.1080/2162402X.2015.1014242 26140237 PMC4485803

[247] Yamauchi Y Safi S Blattner C Rathinasamy A Umansky L Juenger S. Circulating and tumor myeloid-derived suppressor cells in resectable non-small cell lung cancer. Am J Respir Crit Care Med. (2018) 198:777–87. 10.1164/rccm.201708-1707OC 29617574

[248] Yu L Quinn MT Cross AR Dinauer MC. Gp91(phox) is the heme binding subunit of the superoxide-generating NADPH oxidase. Proc Natl Acad Sci USA. (1998) 95:7993–8. 10.1073/pnas.95.14.7993 9653128 PMC20917

[249] Perrot I Michaud HA Giraudon-Paoli M Augier S Docquier A Gros L. Blocking antibodies targeting the CD39/CD73 immunosuppressive pathway unleash immune responses in combination cancer therapies. Cell Rep. (2019) 27:2411–25.e9. 10.1016/j.celrep.2019.04.091 31116985

[250] Linden J Koch-Nolte F Dahl G. Purine release, metabolism, and signaling in the inflammatory response. Annu Rev Immunol. (2019) 37:325–47. 10.1146/annurev-immunol-051116-052406 30676821

[251] Mbongue JC Nicholas DA Torrez TW Kim NS Firek AF Langridge WH. The role of indoleamine 2, 3-dioxygenase in immune suppression and autoimmunity. Vaccines. (2015) 3:703–29. 10.3390/vaccines3030703 26378585 PMC4586474

[252] Ercolano G Garcia-Garijo A Salome B Gomez-Cadena A Vanoni G Mastelic-Gavillet B. Immunosuppressive mediators impair proinflammatory innate lymphoid cell function in human malignant melanoma. Cancer Immunol Res. (2020) 8:556–64. 10.1158/2326-6066.CIR-19-0504 32019778

[253] Mastelic-Gavillet B Navarro Rodrigo B Decombaz L Wang H Ercolano G Ahmed R. Adenosine mediates functional and metabolic suppression of peripheral and tumor-infiltrating CD8^+^ T cells. J Immunother Cancer. (2019) 7:257. 10.1186/s40425-019-0719-5 31601268 PMC6788118

[254] Li J Wang L Chen X Li L Li Y Ping Y. CD39/CD73 upregulation on myeloid-derived suppressor cells via TGF-beta-mTOR-HIF-1 signaling in patients with non-small cell lung cancer. Oncoimmunology. (2017) 6:e1320011. 10.1080/2162402X.2017.1320011 28680754 PMC5486179

[255] Kim HR Park SM Seo SU Jung I Yoon HI Gabrilovich DI. The ratio of peripheral regulatory T cells to Lox-1^+^ polymorphonuclear myeloid-derived suppressor cells predicts the early response to anti-PD-1 therapy in patients with non-small cell lung cancer. Am J Respir Crit Care Med. (2019) 199:243–6. 10.1164/rccm.201808-1502LE 30339766 PMC6835091

[256] Srivastava MK Zhu L Harris-White M Kar UK Huang M Johnson MF. Myeloid suppressor cell depletion augments antitumor activity in lung cancer. PLoS ONE. (2012) 7:e40677. 10.1371/journal.pone.0040677 22815789 PMC3398024

[257] Hoeppner LH Wang Y Sharma A Javeed N Van Keulen VP Wang E. Dopamine D2 receptor agonists inhibit lung cancer progression by reducing angiogenesis and tumor infiltrating myeloid derived suppressor cells. Mol Oncol. (2015) 9:270–81. 10.1016/j.molonc.2014.08.008 25226814 PMC4277897

[258] Zhao Y Shao Q Zhu H Xu H Long W Yu B. Resveratrol ameliorates lewis lung carcinoma-bearing mice development, decreases granulocytic myeloid-derived suppressor cell accumulation and impairs its suppressive ability. Cancer Sci. (2018) 109:2677–86. 10.1111/cas.13720 29959821 PMC6125446

[259] Lechner MG Liebertz DJ Epstein AL. Characterization of cytokine-induced myeloid-derived suppressor cells from normal human peripheral blood mononuclear cells. J Immunol. (2010) 185:2273–84. 10.4049/jimmunol.1000901 20644162 PMC2923483

[260] Liu D You M Xu Y Li F Zhang D Li X. Inhibition of curcumin on myeloid-derived suppressor cells is requisite for controlling lung cancer. Int Immunopharmacol. (2016) 39:265–72. 10.1016/j.intimp.2016.07.035 27497194

[261] Lei X Lei Y Li JK Du WX Li RG Yang J. Immune cells within the tumor microenvironment: biological functions and roles in cancer immunotherapy. Cancer Lett. (2020) 470:126–33. 10.1016/j.canlet.2019.11.009 31730903

[262] Meyer C Cagnon L Costa-Nunes CM Baumgaertner P Montandon N Leyvraz L. Frequencies of circulating MDSC correlate with clinical outcome of melanoma patients treated with ipilimumab. Cancer Immunol Immunother. (2014) 63:247–57. 10.1007/s00262-013-1508-5 24357148 PMC11029062

[263] Tarhini AA Edington H Butterfield LH Lin Y Shuai Y Tawbi H. Immune monitoring of the circulation and the tumor microenvironment in patients with regionally advanced melanoma receiving neoadjuvant ipilimumab. PLoS ONE. (2014) 9:e87705. 10.1371/journal.pone.0087705 24498358 PMC3912016

[264] Weber J Gibney G Kudchadkar R Yu B Cheng P Martinez AJ. Phase I/II study of metastatic melanoma patients treated with nivolumab who had progressed after ipilimumab. Cancer Immunol Res. (2016) 4:345–53. 10.1158/2326-6066.CIR-15-0193 26873574 PMC4818672

[265] Wang PF Song SY Wang TJ Ji WJ Li SW Liu N. Prognostic role of pretreatment circulating MDSCs in patients with solid malignancies: a meta-analysis of 40 studies. Oncoimmunology. (2018) 7:e1494113. 10.1080/2162402X.2018.1494113 30288362 PMC6169582

[266] Clavijo PE Moore EC Chen J Davis RJ Friedman J Kim Y. Resistance to CTLA-4 checkpoint inhibition reversed through selective elimination of granulocytic myeloid cells. Oncotarget. (2017) 8:55804–20. 10.18632/oncotarget.18437 28915554 PMC5593525

[267] Holmgaard RB Zamarin D Lesokhin A Merghoub T Wolchok JD. Targeting myeloid-derived suppressor cells with colony stimulating factor-1 receptor blockade can reverse immune resistance to immunotherapy in indoleamine 2,3-dioxygenase-expressing tumors. EBioMedicine. (2016) 6:50–8. 10.1016/j.ebiom.2016.02.024 27211548 PMC4856741

[268] Mao Y Eissler N Blanc KL Johnsen JI Kogner P Kiessling R. Targeting suppressive myeloid cells potentiates checkpoint inhibitors to control spontaneous neuroblastoma. Clin Cancer Res. (2016) 22:3849–59. 10.1158/1078-0432.CCR-15-1912 26957560

